# Haplotype-Phased Synthetic Long Reads from Short-Read Sequencing

**DOI:** 10.1371/journal.pone.0147229

**Published:** 2016-01-20

**Authors:** James A. Stapleton, Jeongwoon Kim, John P. Hamilton, Ming Wu, Luiz C. Irber, Rohan Maddamsetti, Bryan Briney, Linsey Newton, Dennis R. Burton, C. Titus Brown, Christina Chan, C. Robin Buell, Timothy A. Whitehead

**Affiliations:** 1 Department of Chemical Engineering and Materials Science, Michigan State University, East Lansing, Michigan, United States of America; 2 Department of Plant Biology, Michigan State University, East Lansing, Michigan, United States of America; 3 Michigan Center for Translational Pathology, University of Michigan, Ann Arbor, 48109, United States of America; 4 Department of Microbiology and Molecular Genetics and Department of Computer Science and Engineering, Michigan State University, East Lansing, Michigan, United States of America; 5 BEACON Center for the Study of Evolution in Action, Michigan State University, East Lansing, Michigan, United States of America; 6 Department of Integrative Biology, Michigan State University, East Lansing, Michigan, United States of America; 7 Department of Immunology and Microbial Science, The Scripps Research Institute, La Jolla, California, United States of America; 8 Center for HIV/AIDS Vaccine Immunology and Immunogen Discovery, The Scripps Research Institute, La Jolla, California, United States of America; 9 International AIDS Vaccine Initiative Neutralizing Antibody Center, The Scripps Research Institute, La Jolla, California, United States of America; 10 Population Health and Reproduction, University of California Davis, Davis, California, United States of America; 11 Department of Biosystems and Agricultural Engineering, Michigan State University, East Lansing, Michigan, United States of America; Indiana University, UNITED STATES

## Abstract

Next-generation DNA sequencing has revolutionized the study of biology. However, the short read lengths of the dominant instruments complicate assembly of complex genomes and haplotype phasing of mixtures of similar sequences. Here we demonstrate a method to reconstruct the sequences of individual nucleic acid molecules up to 11.6 kilobases in length from short (150-bp) reads. We show that our method can construct 99.97%-accurate synthetic reads from bacterial, plant, and animal genomic samples, full-length mRNA sequences from human cancer cell lines, and individual HIV *env* gene variants from a mixture. The preparation of multiple samples can be multiplexed into a single tube, further reducing effort and cost relative to competing approaches. Our approach generates sequencing libraries in three days from less than one microgram of DNA in a single-tube format without custom equipment or specialized expertise.

## Introduction

The short-read assembly paradigm currently dominates genomics [1, 2]. However, the loss of linkage information during the generation of short reads limits their utility. In particular, short reads are insufficient to phase the haplotypes of individuals within mixtures of similar sequences, including homeologous and homologous chromosomes in polyploids [3, 4], viral quasispecies [5], multiply or alternatively spliced mRNA [6], genes from metagenomic samples containing related organisms [7, 8], and immune antibody gene repertoires [9]. In these cases, additional information is required to determine whether mutations separated by distances longer than the read length are present in the same individual.

This limitation can be overcome by technologies that provide long continuous sequence reads. “True” long read technologies such as Pacific Biosciences SMRT sequencing and nanopore technologies [10, 11] have proven invaluable in select applications, but require extensive computational error correction [12, 13]. As an alternative, sample preparation protocols have emerged that enable long “synthetic” reads to be constructed from conventional short reads [14–21]. However, these approaches have been limited by generalizability, cost, throughput, or synthetic read length.

Here, we present a library preparation method that overcomes these limitations, providing a general platform for affordable and reliable synthetic read generation from a wide range of input nucleic acid types. In contrast to competing approaches, our method requires no specialized equipment or proprietary software and is carried out in a single tube. We demonstrate generalizability by assembling synthetic reads from a range of samples including genomic DNA from bacteria, plants, and an animal, mRNA isolated from human cancer lines, and mock viral patient samples. We validate 99.97%-accurate synthetic reads up to 11.6 kb in length, and show their utility for improving a plant draft genome assembly. We show that the preparation of multiple samples can be multiplexed into a single tube, further reducing effort and cost relative to competing approaches. Finally, using synthetic reads, we directly observe up to thirty-five splice junctions in an individual mRNA and individual haplotypes from mixtures of highly similar molecules with a low rate of chimera formation.

## Results

**Fig 1:**
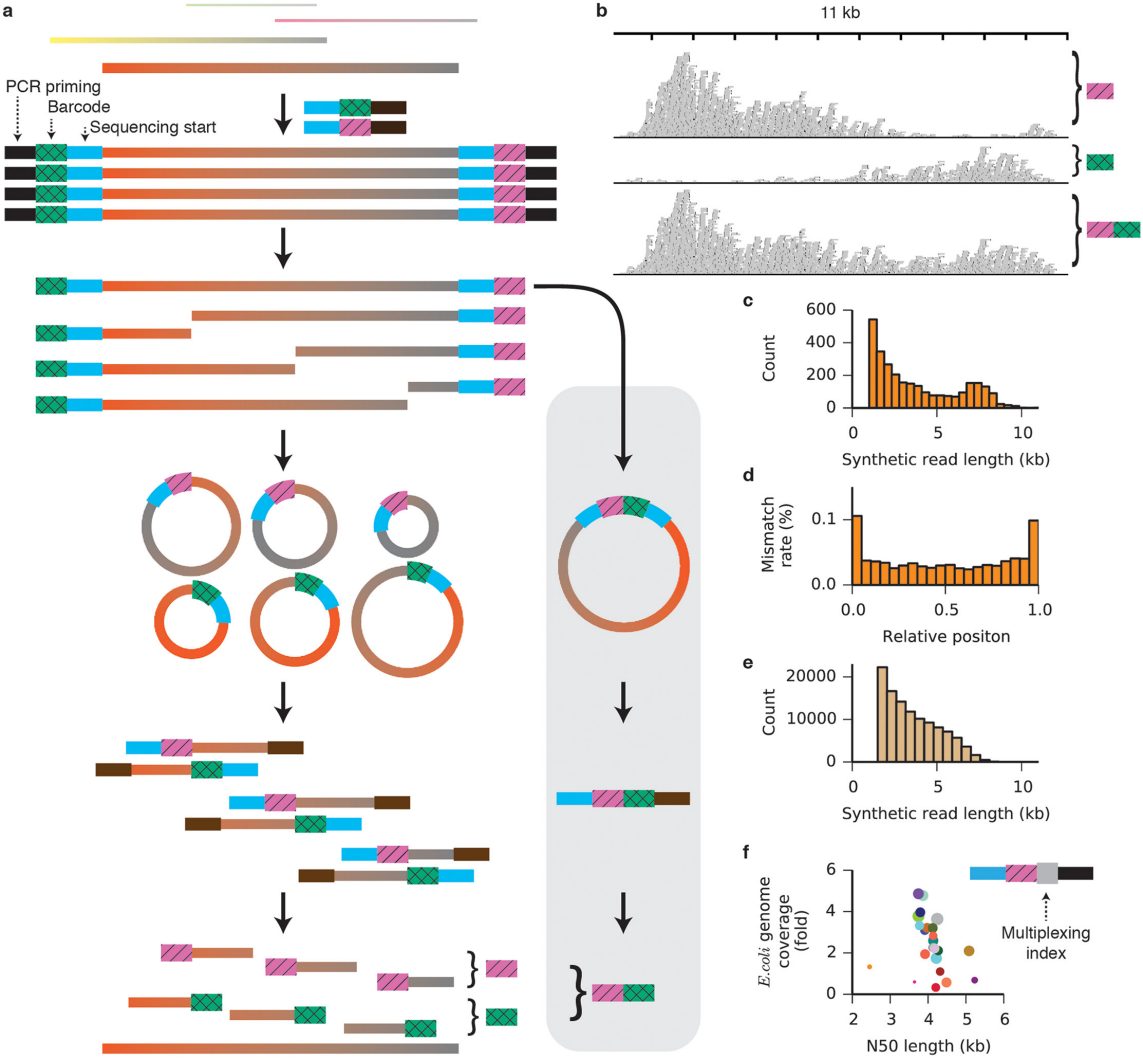
A method for assembling synthetic long reads. (a) Schematic of the approach. A supplemental barcode-pairing protocol (grey box) resolves the two distinct barcodes affixed to each original target molecule. (b) Reads associated with two distinct barcodes are shown aligned to the *E*. *coli* MG1655 reference genome. Barcode pairing merges the groups (bottom), increasing and evening the coverage and allowing assembly of the full 10-kb target sequence. (c) Length histogram of synthetic reads assembled from *E*. *coli* MG1655 genomic reads (minimum length 1 kb). The N50 length of the synthetic reads is 6.0 kb, and the longest synthetic read is 11.6 kb. (d) Mismatch rates of synthetic reads from the *E*. *coli* MG1655 dataset as a function of relative position along the synthetic read. (e) Length histogram of synthetic long reads assembled from *Gelsemium sempervirens* genomic reads (minimum length 1.5 kb). The N50 length of the synthetic reads is 4.3 kb. (f) An additional multiplexing index region (grey square) allows adapter-ligated samples to be mixed and processed in a single tube. Genomic DNA from twenty-four experimentally evolved strains of *E*. *coli* was separately ligated to adapters and amplified, then mixed into a single tube for the remaining steps of the protocol. *E*. *coli* genome coverage and N50 length are plotted for synthetic reads from each strain. Circle size indicates the number of short reads demultiplexed to a given strain.

The approach is illustrated schematically in Fig 1a. DNA fragments of lengths up to 20 kb are appended at each end with adapters containing a degenerate barcode region flanked by defined sequences, such that every target fragment becomes associated with two unique barcodes. PCR with a single primer produces many copies of each target molecule along with its two associated barcodes. The priming sites are removed and a single break (on average) is enzymatically induced in each copy to expose regions of unknown sequence at the newly created ends. Those regions are brought into proximity with the barcode at the opposite end of each fragment by intramolecular circularization (S1 Fig). Next, the molecules are linearized and a sequencing-ready library compatible with the Illumina platform is prepared (S2 Fig). The resulting short reads begin with the barcode sequence and continue into the unknown region. Following sequencing, reads are grouped by common barcodes. The two distinct barcodes appended to each target molecule can be identified and paired (Fig 1a and S3 Fig), allowing the two barcode-defined groups derived from the two ends of the original fragment to be combined. The read groups are assembled independently and in parallel to reconstruct the full sequences of the original DNA molecules.

To validate the accuracy of the synthetic long reads, we prepared sequencing libraries from genomic DNA isolated from *E*. *coli* MG1655. Illumina 150-bp paired-end reads were trimmed to remove barcodes, adapter sequences, and regions of low quality, and sorted into barcode-delineated groups. When one such group was aligned to the MG1655 reference genome, 94.7% of the reads aligned to the same 11-kb region. The coverage distribution across the region was non-uniform, dropping off with distance from the barcoded end (Fig 1b). A total of 1,215 barcode pairs were identified and their read groups merged. Because coverage from one barcode is high in the region of the target molecule where coverage from its partner is low, merging groups not only increases but also evens the coverage across the target. After barcode pairing, 2,792 read groups contained at least fifty read pairs. Independent *de novo* assembly of each group yielded 2,878 synthetic reads of length greater than 1 kb (Fig 1c, S1 Table), with the longest reaching 11.6 kb. Barcode pairing improved the N50 synthetic read length (defined throughout this work such that half of the total bases in synthetic reads longer than 1 kb are in synthetic reads longer than the N50 synthetic read length) and reduced the number of redundant synthetic reads (S4 and S5 Figs). To determine the fidelity of assembly, synthetic reads longer than 1.5 kb were aligned to the MG1655 reference genome [22]. The mismatch rate within the aligned regions of the synthetic reads was 0.04% (S2 Table). Errors were more common at the ends of the synthetic reads, where short-read coverage was low (Fig 1d and S6 Fig). When 100 nucleotides were trimmed from each end of the synthetic reads, the mismatch rate dropped to 0.03%. Transition mutations (C/T and A/G) made up 75.6% of all mismatches, suggesting that the PCR amplification step is the dominant source of error [23]. Although it may be that GC-rich regions of the genomes are underrepresented in the synthetic reads, we did not detect GC bias in the synthetic reads relative to the overall GC content of the genome (S7 Fig).

We further evaluated the accuracy of our method with genomic DNA isolated from higher organisms with well-developed draft genome assemblies. From *Gallus gallus* (chicken) genomic DNA [24], we collected 103,601,271 paired-end 150-bp reads (S1 Table), from which we assembled 125,203 synthetic reads longer than 1 kb, with an N50 length of 2.0 kb. The length distribution (S8 Fig) and low N50 length relative to the shearing length indicate that this library was under-sequenced, and additional sequencing would yield longer synthetic reads. Nonetheless, all but 661 (0.13%) of the 510,070 synthetic reads of all lengths aligned to the *G*. *gallus* reference genome. Additionally, we generated 1,411 synthetic reads longer than 1 kb (N50 of 3.1 kb) from a doubled-monoploid potato (*Solanum tuberosum* Group Phureja) [3, 25] (S8 Fig); 94.2% of the synthetic reads aligned to the draft reference genome (S3 Table).

Having validated the method, we used synthetic long reads to improve a shotgun *de novo* assembly of the genome of *Gelsemium sempervirens*, an ornamental and medicinal plant species with an estimated genome size of 312 Mb. A draft assembly was first created from 161.4 million Illumina whole-genome shotgun paired end reads. We then prepared a barcoded library and assembled 111,054 synthetic reads longer than 1.5 kb (Fig 1e and S9 Fig), with an assembly N50 length of 4.3 kb (S1 Table). A total of 397.8 Mb of synthetic reads (S4 Table) were used to scaffold the assembly. Incorporation of synthetic reads improved the assembly from 25,276 contigs with an N50 contig length of 19,656 bp to 18,106 scaffolds with an N50 scaffold length of 29,078 bp (Table 1). Maximum contig length also increased, from 198 kb in the shotgun assembly to 366 kb in the synthetic read assembly. Assembly quality metrics generated with the CEGMA pipeline (S5 Table) and by alignment of cleaned RNA-seq reads (S6 Table) were consistent with an increased representation of the genome after incorporation of the synthetic long reads.

**Table 1:** Genome assembly statistics for *G*. *sempervirens*.

	Shotgun contigs	Synthetic read scaffolds
Contig/scaffold N50 size (bp)	19,656	29,078
Total assembly size (bp)	215,038,998	218,719,799
No. of contigs/scaffolds	25,276	18,106
Maximum length (bp)	197,779	365,589

Small contigs (< 1 kb) were filtered out.

In contrast to dilution-based synthetic read approaches [17–19], the intramolecular circularization step in our protocol makes it possible to prepare a library in a single tube. We further exploited this property to allow multiple samples to be combined and prepared in the same mixture, yielding considerable savings in cost and time. To accomplish this, we modified the adapters to include multiplexing index sequences between the PCR priming region and the molecule-specific barcode (Fig 1f, S10 Fig). Genomic DNA from each of twenty-four laboratory-evolved *E*. *coli* strains [26] was isolated, sheared to 6–10 kb, ligated to adapters containing both a multiplexing index unique to each strain and a molecule-specific barcode, and amplified by PCR. Purified PCR products were then mixed and the remainder of the library preparation protocol was performed on the single mixed sample. Sequencing reads were demultiplexed by project according to standard 6-bp index read, then further demultiplexed by strain according to the barcode-adjacent multiplexing index identified in the forward read, sorted by barcode, and assembled in parallel (S11 Fig, S7 Table). The summed lengths of the synthetic reads longer than 1 kb exceeded twofold genome coverage for sixteen out of the twenty-four strains, with a median genome coverage of 2.3 fold and median N50 of 4.1 kb. Cases of low coverage resulted from either adding insufficient amplified DNA into the multiplexed mixture (small circles, Fig 1f) or carrying too few doubly barcoded molecules into the amplification step (large circles with high N50 but low coverage, Fig 1f). Along with the nanogram-level input requirements, single-tube format, and competitive assembly length metrics demonstrated earlier, single-tube multiplexing provides a substantial advantage in throughput, convenience, and cost over competing synthetic read techniques for genome assembly and phasing (S8 Table).

**Fig 2:**
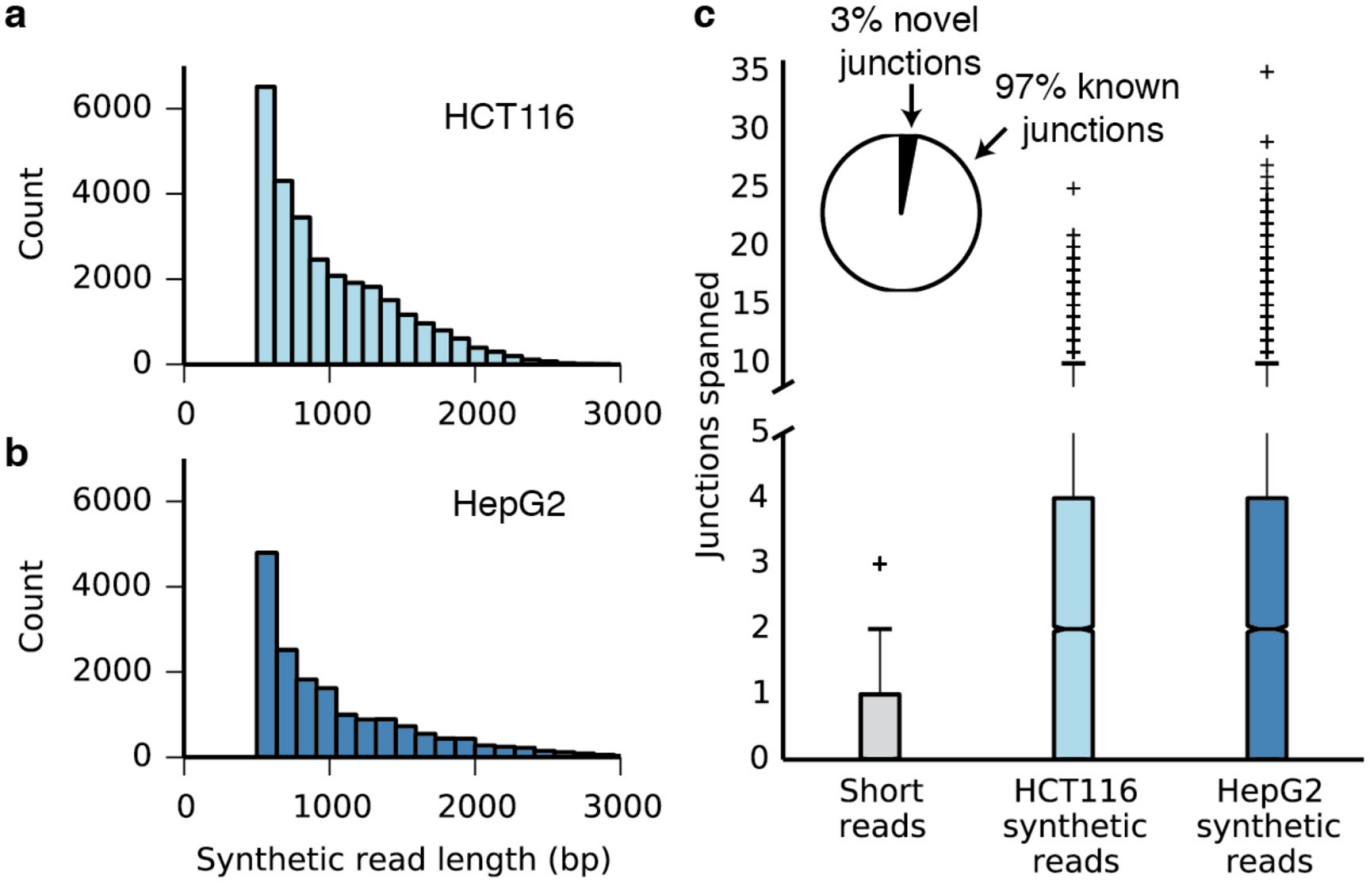
Individual assembly of full-length mRNA sequences. (a) Length distribution of synthetic long reads (minimum length 500 bp) from HCT116 mRNA. (b) Length distribution of synthetic long reads (minimum length 500 bp) from HepG2 mRNA. (c) Box plots showing the number of splice junctions spanned by short reads and synthetic long reads. The axis is broken between 5–10 junctions spanned and the scale changed; a version with a standard axis is presented as S12 Fig. Inset: 97% of the junctions identified in the synthetic reads are known, providing validation for the method.

We next asked whether our method could be extended to applications beyond genome assembly and phasing. In RNA-seq experiments, the presence of transcripts resulting from multiple splicing events must be inferred statistically because individual reads are too short to regularly span multiple splice junctions. We used synthetic long reads to directly observe multiply spliced messenger RNA isolated from human cancer cells. We modified the Smart-seq2 method [27] to incorporate adapters with molecule-specific barcodes during the reverse transcription and second-strand synthesis steps (S12 Fig). The barcoded cDNA product was amplified, broken, circularized, and prepared for sequencing. From mRNA isolated from HCT116 and HepG2 cells, we assembled 28,689 and 16,929 synthetic reads, respectively, of lengths between 0.5 and 4.6 kb (Fig 2). Approximately 97% of the splice junctions captured by the synthetic long reads have been observed previously (Fig 2c, S9, S10 and S11 Tables). In contrast to conventional RNA-seq reads, synthetic reads spanned multiple splice junctions (Fig 2c, S13 Fig), with a median of 2.0 spanned junctions per synthetic read for both samples and a maximum of 35 spanned junctions. Examination of the synthetic reads revealed examples of differential splicing between the HCT116 and HepG2 cell lines, as well as a novel transcript in the HCT116 cell line (S14 Fig). Notably, our protocol can be adapted for single-cell RNA-seq methods, potentially allowing discrimination of multiple splice variants for individual cells.

Highly accurate haplotype-phased sequences open new routes to studying mixtures of similar yet distinct individuals. These include viral quasispecies, which consist of near-identical genomes harboring key mutations, isolated B-cells, whose genomes encode therapeutic antibodies differing mainly in a few hypervariable regions, and environmental samples, which contain closely related strains with homologous genes and operons. In particular, the ability to generate thousands of complete, individual, haplotype-phased viral genome sequences would provide an unprecedented view of the ways viruses adapt to immune responses and environmental changes such as the introduction of antiretroviral therapies. The TruSeq Synthetic Long Reads approach is incompatible with mixtures of similar molecules because it assigns a barcode to a diluted pool of a few hundred target molecules. When drawn from a large genome these molecules are unlikely to overlap, but when all molecules are similar each must be diluted into its own well, limiting throughput.

**Fig 3:**
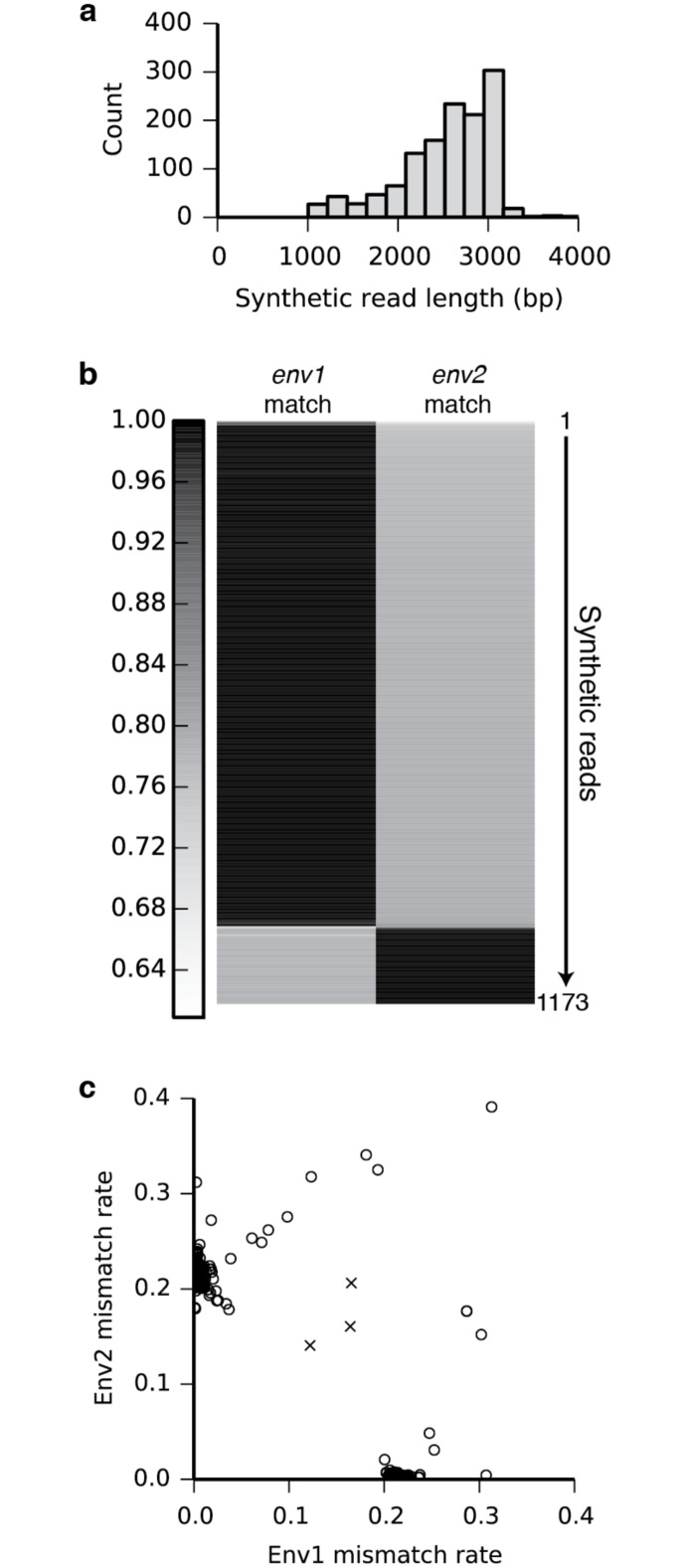
Individual assembly of full-length *env* genes from a mixture of two variants. (a) The length distribution of the synthetic long reads (minimum length 1 kb) shows assembly of full-length 3-kb *env* gene sequences. (b) 1,173 synthetic reads between 1.5 and 3.2 kb in length were aligned to each of the two original *env* sequences (*env1* and *env2*). The alignment match rates are shown as a heatmap, with each synthetic read represented by a thin horizontal line. The majority of the synthetic reads align with low error to exactly one of the two original sequences, indicating high accuracy and a low rate of chimera formation. Chimeric reads would be expected to match both original sequences at intermediate accuracies. (c) Scatter plot showing the mismatch rates of each synthetic read against the two known *env* sequences. Synthetic reads (open circles to emphasize extensive overlap) cluster into two distinct groups along the axes (near-zero mismatch rate). Even the sixteen reads that do not fall on the clusters are distant from three manually created mock chimeras (crosses), indicating a low frequency of chimera formation.

To demonstrate the ability to resolve and haplotype individual genes, we first generated barcoded reads from a mixture of plasmids. As expected, each barcode-defined group of reads was dominated by sequences unique to a single parent (S15 Fig). Next, we sequenced a mock mixture of two *env* gene variants. *Env* is a 3-kb HIV gene that encodes the envelope glycoprotein, which triggers infection by binding CD4 and a co-receptor (either CXCR4 or CCR5) and is the primary target for HIV vaccine development [28]. 712 out of the 1,280 *de novo*-assembled synthetic long reads approached the expected full length of 3 kb (Fig 3a). We were able to unambiguously identify 1,157 out of 1,173 (98.6%) assembled and cleaned sequences between 1.5 and 3.2 kb in length as one or the other of the two variants. The sixteen outlier reads that do not map to one of the two clusters (Fig 3c) have high mismatch rates against both parent sequences, rather than intermediate mismatch with both as would be expected of chimeric sequences. These results indicate accurate assembly and minimal chimera formation.

**Fig 4:**
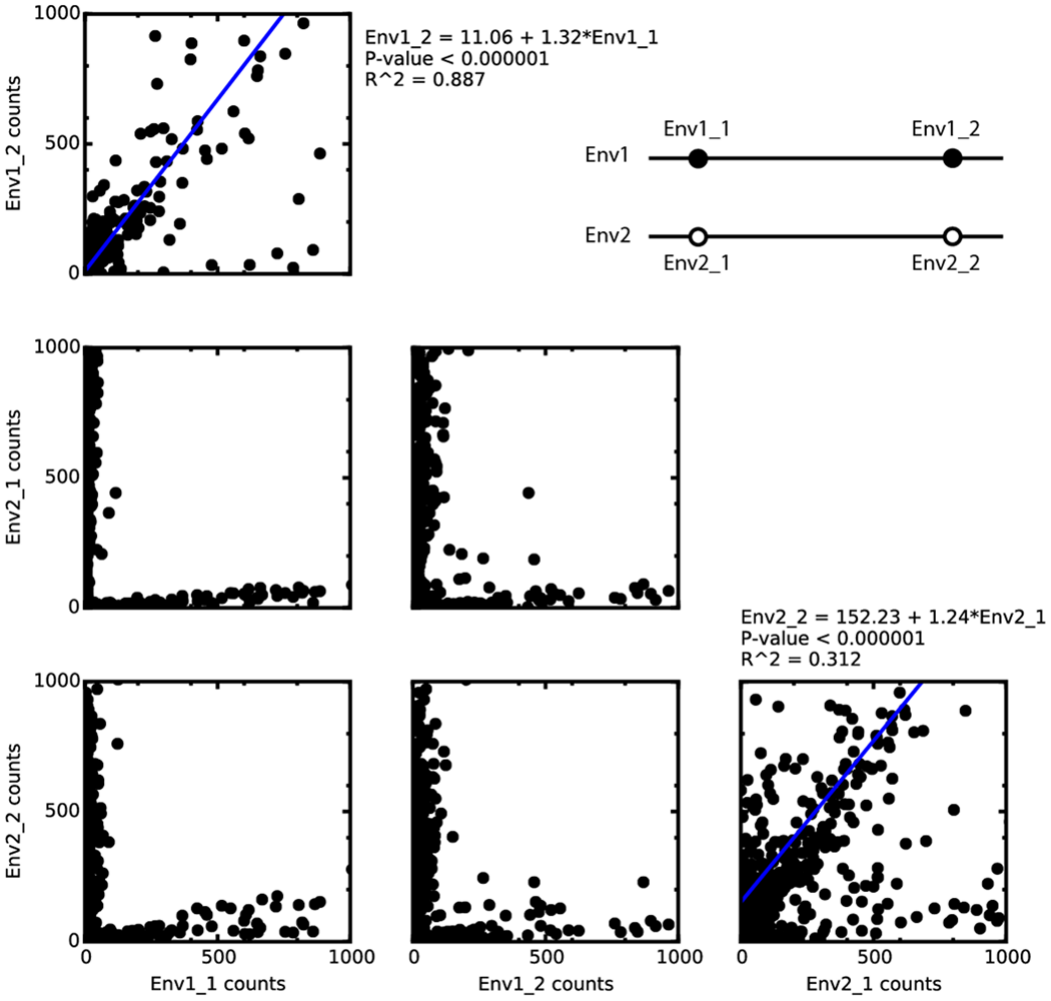
Simulated haplotype phasing by correlation of unique sequences within barcode-defined groups. Short unique sequences were identified at each end of the two variants (Env1_1 and Env1_2 from variant 1, Env2_1 and Env2_2 from variant 2). Each barcode-defined group of short reads was searched for the four sequences. A high number of counts of occurrences of a unique sequence from near the 5’ end of one *env* variant (Env1_1, Env2_1) in a barcode-defined group of short reads is a strong predictor of a high number of occurrences of a second unique sequence from the 3’ end of the same variant (Env1_2, Env2_2) in the same group, and also a strong predictor of a low number of occurrences of the unique sequence from the 3’ end of the other variant. Therefore, the haplotype across these two loci in a given barcoded individual can be phased regardless of the length or identity of the intervening sequence.

Unfortunately, the two variants used in this experiment were distinct enough that each 150-bp window across the gene contained polymorphisms. Non-barcoded short reads could therefore unambiguously assemble and haplotype both versions of the full gene. To demonstrate our method’s ability to haplotype variants at distant loci without relying on polymorphisms to bridge across intervening sequence, we chose unique sequences from each end of each variant (Env1_1 and Env1_2 from variant 1; Env2_1 and Env2_2 from variant 2) and counted the number of times these sequences appeared in each barcode-defined group of unassembled short reads. Linear regression analysis (Fig 4) showed that within each barcode group, a large number of short reads containing one sequence from one variant is a strong predictor (R^2^ = 0.887 for Env1_1 and Env1_2; R^2^ = 0.312 for Env2_1 and Env2_2) of a large number of reads with the other sequence from the same variant, and of relatively low numbers of reads with the sequences from the other variant. Because short reads mapping to the intervening regions were not considered, there was no opportunity for polymorphisms in these regions to bridge short reads from one site to the other. The correlations between correct pairs confirm the fidelity of the barcode-mediated read grouping, and demonstrate that even if the intervening sequence (which is much longer than the read length) were identical, barcoded reads would allow haplotype phasing at distant loci.

## Discussion

We have demonstrated a versatile approach to assemble individual DNA sequences from mixed-population short-read data from a variety of DNA and RNA samples. Our approach offers important advantages over competing synthetic read methods (S8 and S12 Tables). Many samples can be multiplexed and prepared for sequencing in three days in a single tube with no custom equipment or specialized expertise, providing significant benefits in cost and throughput. Our method can be applied to a wider range of sample types than competing technologies, from genomic fragments to uniform mixed populations, and its novel barcode pairing protocol yields longer synthetic reads than previous circularization-based approaches.

Although our circularization approach is conceptually similar to mate pair protocols, which are useful for genome scaffolding and haplotyping, synthetic reads have distinct advantages in many applications. Importantly, while mate pairs can arrange contigs into scaffolds with intervening gaps of estimated length, synthetic long reads fill in the gaps with complete and highly accurate sequence. While in this report we have used an off-the-shelf assembler to construct synthetic reads, our method is fully compatible with emerging alternative approaches that use unassembled barcoded reads, or “read clouds,” to maximize haplotyping information [29].

Recently, the utility of long reads for mRNA analysis has been demonstrated by studies using Pacific Biosciences [30] and TruSeq Long Reads [31]. When long reads are not available, statistical methods are widely used to infer the presence of mRNA isoforms from short-read RNA-seq data [32, 33]. Isoform inference relies firstly on reads that span splice junctions, which are reliable but are limited by read length, and secondly on read pairs that span splice junctions (one read from the pair maps entirely to one exon and the other read maps to a second exon), which depend on estimates of insertion size and hypotheses of possible isoforms. Different statistical approaches incorporate these data using different statistical hypotheses, algorithms, and assumptions. In contrast, synthetic reads directly observe the isoform, providing an unambiguous measurement without relying on inference of any kind. Furthermore, synthetic reads can measure long-range association between exons at distances larger than the insert size, which is not possible for even a perfect inference algorithm.

Like all sequencing approaches, our method comes with caveats. We have shown that coverage is uneven across barcode groups, varying by up to two orders of magnitude (S9 Fig). As a result of their common reliance on long-range PCR, our method and the TruSeq Long Reads approach may exhibit bias against regions of the genome due to secondary structure or GC content, though we do not observe GC bias in the datasets presented here (S7 Fig). Synthetic read methods also fail to assemble regions containing short repeats, which can be accurately sequenced by true long read methods (McCoy et al. 2014).

Because synthetic reads are built from overlapping short reads, improved read length and accuracy come at a cost of sequencing depth. In the examples presented here, between 43 and 225 nucleotides of short-read sequencing were used to generate each nucleotide of synthetic read (S1 Table). The cost of synthetic long reads is justified by additional linkage information, improved accuracy, and lowered resource demand through parallelization of the computational pipeline. Synthetic reads will become increasingly attractive as sequencing throughput increases and costs drop, further justifying the exchange of quantity for quality.

## Materials and Methods

### Library preparation for synthetic long read assembly

A 100 μL solution of two oligonucleotides (oligos 1 and 2, S13 Table, at 2 uM and 5 uM, respectively) in NEB buffer 2 (New England Biolabs, Ipswich, MA) was heated to 95°C for 10 minutes and allowed to cool slowly to 37°C. 5 units of Klenow exo- (NEB) and 0.3 mM each dNTP (NEB) were added and the mixture was incubated at 37°C for 60 minutes. The DNA to be sequenced was typically diluted to 50 μL at 10 ng/μL and fragmented into ~10 kb pieces with a g-TUBE (Covaris, Woburn, MA) by centrifugation at 4200 g according to the manufacturer’s protocol. The DNA was end-repaired with the NEBNext End Repair Module (NEB) according to the manufacturer's protocol, purified with a Zymo column (Zymo Research, Irvine, CA) and eluted in 8 μL buffer EB (Qiagen, Hilden, Germany). The DNA was then dT-tailed by incubation in 1X NEB buffer 2 with 1 mM dTTP (Life Technologies, Carlsbad, CA), 5 units Klenow exo-, and 10 units polynucleotide kinase (NEB) at 37°C for 1 hour. 250 fmol of library DNA (4 μL) and 5 pmol of barcode adapters (1 μL) were ligated with 5 μL TA/Blunt MasterMix (NEB) according to the manufacturer's protocol, gel purified with size selection with the Qiagen Gel Extraction kit if necessary, and eluted in 50 μL buffer EB. The concentration of doubly ligated product was determined by qPCR and/or dilution-series PCR. The library was diluted to about 100,000 doubly ligated molecules per μL. Approximately 100,000 molecules of adapter-ligated DNA were amplified by PCR in 150 μL reactions split across eight PCR tubes with LongAmp Taq DNA polymerase (NEB) using a single primer (oligo 2, S13 Table) [34, 35] at 0.5 mM and the following thermocycling conditions: 92°C for 2 minutes, followed by 40 cycles of 92°C for 10 seconds, 55°C for 30 seconds, and 65°C for 3 minutes/kb, followed by a final hold at 65°C for 10 minutes.

The PCR product was gel purified with the Qiagen Gel Extraction kit, eluted in 50 μL of buffer EB, and quantified by absorbance at 260 nm. 200 ng to 1 μg of DNA were mixed with 1 unit of USER enzyme (NEB) in a 45 μL reaction volume and incubated for 30 minutes at 37°C. 100 ug/mL bovine serum albumin and 5 μL of dsDNA fragmentase buffer were added and the mixture incubated on ice for 5 minutes. 0.5–2 μL of dsDNA fragmentase (NEB) (volume adjusted based on amount and length of DNA to be fragmented) were added and the mixture was incubated at 37°C for 15 minutes. The reaction was stopped by addition of 5 μL of 0.5 M EDTA and fragmentation was confirmed by the presence of a smear on an agarose gel. The DNA was purified with 0.8 volumes of Ampure XP beads (Beckman Coulter, Brea, CA), eluted in 20 μL buffer EB, and quantified by absorbance at 260 nm. 2 μL of 10X NEB Buffer 2 were added and fragmented DNA was incubated with 0.5 μL of *E*. *coli* DNA ligase (NEB) for 20 minutes at 20°C. 3 units T4 DNA polymerase, 5 units Klenow fragment (both from NEB), and 50 μM biotin-dCTP (Life Technologies) were added and the reaction was incubated for 10 minutes at 20°C. 50 μM dGTP, dTTP, and dATP were added and the mixture was incubated for an additional 15 minutes, purified with 1 volume of Ampure XP beads, eluted in 20 μL buffer EB, and quantified by absorbance at 260 nm. 200–1000 ng of DNA at a final concentration of 1 ng/μL were mixed with 3000 units of T4 DNA ligase and T4 DNA ligase buffer to 1X, and incubated at room temperature for 16–48 hours.

Linear DNA was digested by addition of 10 units of T5 exonuclease and incubation at 37°C for 60 minutes. Circularized DNA was purified with a Zymo column and eluted in 130 μL buffer EB. The DNA was fragmented with the Covaris S2 disruptor to lengths ~500 bp. 20 μL of Dynabeads M-280 Streptavidin Magnetic Beads (Life Technologies) were washed twice with 200 μL of 2X B&W buffer (1X B&W buffer: 5 mM Tris-HCl (pH 7.5), 0.5 mM EDTA, 1 M NaCl) and resuspended in 100 μL of 2X B&W buffer. The DNA solution was mixed with this bead solution and incubated for 15 minutes at 20°C. The beads were washed twice with 200 μL of 1X B&W buffer and twice in 200 μL of Qiagen buffer EB. At this point, 15% (30 μL) of the beads were removed to a new tube for two-tube barcode pairing (see below). The remaining beads were resuspended in NEBNext End Repair Module solution (42 μL water, 5 μL End Repair Buffer, and 2.5 μL End Repair Enzyme Mix), incubated at 20°C for 30 minutes, and washed twice with 200 μL of 1X B&W buffer and twice with 200 μL of buffer EB. The beads were then resuspended in 17 μL water, 2 μL NEB buffer 2, 0.5 μL 10 mM dATP, and 5 units Klenow exo- polymerase, incubated at 37°C for 30 minutes to add dA tails to the DNA fragments, and washed twice with 200 μL of 1X B&W buffer and twice with 200 μL of buffer EB. A 15 micromolar equimolar mixture of two oligonuleotides (oligos 3 and 4, S13 Table) in 1X T4 DNA ligase buffer was incubated at 95°C for 2 minutes and allowed to cool to room temperature over 30 minutes. The beads were resuspended in a solution consisting of 5 μL of NEB Blunt/TA ligase master mix, 0.5 μL of 15 micromolar adapter oligo solution, and 4 μL of water. The mixture was incubated for 15 minutes at room temperature. The beads were washed twice with 200 μL of 1X B&W buffer and twice with 200 μL of buffer EB. For amplification by limited-cycle PCR (lcPCR), the beads were resuspended in a 50 μL PCR solution consisting of 36 μL of water, 10 μL of 5X Phusion HF DNA polymerase buffer, 1.25 μL of each of 10 micromolar solutions of Illumina Index and Universal primers (oligos 5 and 6, S13 Table), and 0.02 units/μL Phusion DNA polymerase (Thermo Fisher Scientific, Waltham, MA). The following thermocycling program was used: 98 degrees for 30 seconds, followed by 18 cycles of 98°C for 10 seconds, 60°C for 30 seconds, and 72°C for 30 seconds, and a final hold at 72 degrees for 5 minutes. The supernatant was retained and the beads discarded. The PCR product was purified with 0.7 volumes of Ampure XP beads and eluted in 10 μL buffer EB, or 500–900 bp fragments were size-selected on an agarose gel, gel-purified with the Qiagen MinElute Gel Extraction kit, and eluted in 15 μL of buffer EB. The size distribution of the DNA was measured with an Agilent bioanalyzer and cluster-forming DNA was quantified by qPCR. The DNA fragments were sequenced on an Illumina MiSeq, NextSeq, or HiSeq with standard Illumina primer mixtures.

### Two-tube barcode pairing

See S1 Note and S3 Fig. Bead-bound DNA was digested with 10 units of SapI in 1X CutSmart buffer in a 20 μL total volume for 1h at 37C. The beads were washed three times with 200 μL of 1X B&W buffer and twice with 200 μL of buffer EB. A 15 μM equimolar mixture of two oligonucleotides (oligos 7 and 8, S13 Table) in 1X T4 DNA ligase buffer was incubated at 95°C for 2 minutes and allowed to cool to room temperature over 30 minutes. The beads were resuspended in a solution consisting of 5 μL of NEB Blunt/TA ligase master mix, 0.5 μL of 15 μM adapter oligo solution, and 4 μL of water. The mixture was incubated for 15 minutes at 4°C and 15 minutes at 20°C. The beads were washed twice with 200 μL of 1X B&W buffer and twice with 200 μL of buffer EB. For amplification by limited-cycle PCR, the beads were resuspended in a 50 μL PCR solution consisting of 36 μL of water, 10 μL of 5X Phusion HF DNA polymerase buffer, 1.25 μL of each of 10 μM solutions of two primers (oligos 6 and 9, S13 Table, with oligo 9 selected to have a different multiplexing index than oligo 5 used above), and 0.02 units/μL Phusion DNA polymerase (Thermo Fisher Scientific). The following thermocycling program was used: 98°C for 30 seconds, followed by 18 cycles of 98°C for 10 seconds, 60°C for 30 seconds, and 72°C for 30 seconds, and a final hold at 72°C for 5 minutes. The supernatant was retained and the beads discarded. DNA was purified with 1.8 volumes of Ampure XP beads and eluted in 10 μL buffer EB. The expected product size of ~170 bp was confirmed by agarose gel electrophoresis and Agilent bioanalyzer. Cluster-forming DNA was quantified by qPCR. The DNA fragments were mixed with the main library so as to comprise 1–5% of the total molecules, and sequenced on an Illumina MiSeq, NextSeq, or HiSeq with standard Illumina primer mixtures.

### Single-tube barcode pairing

See S1 Note, S15 Fig. Two different versions of oligo 1 (S13 Table) were mixed, extended with oligo 2 (S13 Table), and ligated to dT-tailed target fragments as above. The library preparation protocol was carried out as above, except that the extra barcode-pairing steps were omitted. Limited-cycle PCR was performed with 1.25 μL of a 10 micromolar solution oligo 10 in addition to oligos 5 and 6 (S13 Table).

### Complexity determination

A key step in the protocol is the quantification of doubly barcoded fragments prior to PCR. Doubly barcoded fragment concentration in this study was estimated in three ways: quantitative PCR with a quenched fluorescent probe (probe 1, S13 Table), dilution series endpoint PCR, and quantification by next-generation sequencing. For the latter, barcoded molecules were purified and serially diluted. Four dilutions were amplified with oligo 6 and four versions of oligo 11 (S13 Table) containing different multiplexing index sequences. The resulting products were mixed and sequenced with 50-bp single-end reads on an Illumina MiSeq. Reads were demultiplexed and unique barcodes at each dilution were counted. When combined with the multiplexed library preparation strategy, which enables further demultiplexing on the basis of an index in the forward read, many samples can be quantified in a single MiSeq run.

### Library preparation for synthetic long read assembly from mRNA samples

Cell lines HCT 116 (ATCC^®^ CCL-247^™^) and C3A [HepG2/C3A, derivative of Hep G2 (ATCC HB-8065)] (ATCC^®^ CRL-10741^™^) were obtained from ATCC (Manassas, VA). Full-length reverse transcripts were prepared essentially as in [36], with modified primers. The “oligo-dT primer” and “TSO primer” were replaced by oligo 12 and oligo 13 (S13 Table**)**, respectively. Barcoded full-length reverse transcripts were then processed and sequenced as above, starting from the library quantification step.

### Mulitplexed sample preparation

Two *E*. *coli* strains were isolated from each of the twelve recombination treatment populations in Souza *et al*. [26]. Genomic DNA was isolated from each of the twenty-four strains, sheared, end-repaired, and dT-tailed as described above in separate tubes. Twenty-four barcode adapters (S14 Table), identical except for distinct 6-bp multiplexing index regions adjacent to the barcode sequence, were prepared and ligated to the genomic fragments as described above. Adapter-ligated DNA was PCR amplified as above. Purified PCR products were quantified and equal amounts were combined into a single mixture. This mixture was prepared for sequencing following the remaining steps of the above protocol.

### Assembly of synthetic long reads

Barcoded short reads were assembled into synthetic long reads with custom python scripts, available for download at www.github.com/jstapleton/synthetic_reads. In the first step, low-quality regions and barcode sequences are removed using Trimmomatic (v 0.30) [37] and overlapping paired-end reads are merged with FLASh (v 1.2.10) [38]. In the second step, reads are further trimmed and sorted into groups according to their barcodes. In the third step, each group is *de novo* assembled independently with the SPAdes assembler (v 3.1.1) [39].

### Mismatch rate calculation

Synthetic long reads 1500 bp in length or longer assembled from *E*. *coli* K12 MG1655 genomic sequencing reads were aligned to the reference genome^20^ using BWA-MEM (v 0.7.12)^33^. The resulting SAM file was parsed with a custom script to extract mismatch, insertion, deletion, and clipping frequencies.

### Alignment of synthetic reads to the *S*. *tuberosum* assembly

Potato synthetic reads were trimmed by removing 100 nt from each end and then removing reads shorter than 1 kb. The trimmed synthetic reads were then aligned to the *S*. *tuberosum* Group Phureja DM1-3 pseudomolecules (v 4.03) [3, 25] with BWA-MEM (v 0.7.12) [40]. A custom Perl script was used to bin the alignments based on the mapping quality score for the alignment.

### Genome assembly of *G*. *sempervirens*

DNA was isolated from *G*. *sempervirens* (“Carolina Jessamine”) using the CTAB method [41]. DNA was sheared to 300, 500, and 700 bp using a Covaris S2 instrument, end repaired using NEBNext End Repair Module (New England Biolabs, Ipswich, MA) and dA-tailed using Klenow Fragment (New England Biolabs) prior to ligation with annealed universal adaptors. The ligated DNA fragments were size selected using the Agencourt AmPure XP beads (Beckman Coulter, Indianapolis, IN), and then amplified with indexed primers for eight cycles with HiFi HotStart DNA polymerase (KAPA Biosystems, Wilmington, MA). Libraries were gel purified, pooled, and sequenced to 100 nucleotides in the paired end mode on an Illumina HiSeq 2500 instrument. Read quality was assessed using FastQC (v 0.11.2; http://www.bioinformatics.babraham.ac.uk/projects/fastqc/) and adapter sequences were removed and the reads quality trimmed using Cutadapt software (v 1.4.1) [42] using a quality cutoff 20 and a minimum length of 81. *De novo* genome assembly was performed using Velvet (v 1.2.10) [43] using a k-mer length of 51. Contigs shorter than 1,000 bp were filtered out and the remaining contigs were used for downstream analyses.

The *G*. *sempervirens* assembled contigs were scaffolded using the synthetic long reads longer than 1499 bp and the SSPACE-LongRead tool (v1-1) [44]; the default alignment options and scaffolding options were used. Genome assembly quality was evaluated using CEGMA [45] and representation of RNA-sequencing reads (see below).

### *G*. *sempervirens* transcriptome analyses

For RNA-seq analysis, total RNA was extracted from five tissues (immature leaf, stem, stamens, pistils and petal) of *G*. *sempervirens* using the Qiagen RNeasy kit. RNA-seq libraries were constructed using the Kapa library preparation kit (Kapa Biosystems, Wilmington, MA) and sequenced on an Illumina HiSeq 2500 (100 bp, paired end). Read quality assessment, adapter removal, and quality trimming was performed as described for genomic DNA sequences. Cleaned RNA-seq reads were aligned to the *G*. *sempervirens* genome assemblies using TopHat (v 1.4.1) [46] in the single-end mode allowing a maximum of two mismatches; for reporting multiple mapping reads, up to 20 alignments were permitted. Alignment statistics were obtained using SAMtools (v 0.1.19) [47].

### Human mRNA splicing analysis

The assembled long read sequences were processed to remove all poly-A reads, then aligned to hg19/GRCh37 with STAR version 2.3.1z [48] and with GMAP version 2014-12-21 [49]. BEDTools version 2.18.2 [50] was used to count the number of reads mapped to each human gene, and Cufflinks version 2.1.1 [32] was used to quantify gene expression level. The gene annotations used in the analysis are from Ensemble (GRCh37.72). To identify splice junctions from the data, reads that uniquely aligned to the human genome were extracted, and RSEQC version 2.6.1 [51] was used to determine the locations where reads were split and to compare the resulting sets with known splice sites from GRCh37.72.

### Barcode fidelity determination in a plasmid mixture

Six plasmids were mixed, linearized by restriction enzyme digestion, and sequenced. Three of the six plasmids were BioBrick backbones differing only in their antibiotic resistance genes (pSB1C3, pSB1K3, and pSB1A3). The remaining three were pJexpress414 (DNA2.0, Menlo Park, CA) containing different variants of the *E*. *coli* outer membrane protein OmpA. Reads were sorted by barcode, and each bin of reads was searched for short sequences unique to each of the six plasmids. Unique sequence counts for the three OmpA plasmids were plotted for each barcode group in S14 Fig.

### Cleaning of spurious synthetic reads in *env* analysis

Because the *env* samples were sequenced to high coverage, reads containing sequencing errors in the barcode region were abundant enough that truncated synthetic reads were assembled from the reads associated with spurious barcodes. These synthetic reads were removed before further analysis by identifying barcodes with a Hamming distance from another barcode of one or two, and discarding the barcode with the shorter synthetic read. Surviving synthetic reads longer than 1.5 kb were aligned against the two known variant sequences using the EMBOSS (v 6.6.0) *water* local alignment software [52]. Three *env*1/*env*2 chimeras were manually created by cutting and pasting together sequences from the two parents, and subjected to the same analysis.

### Code availability

Scripts used to assemble and analyze synthetic reads are available at https://github.com/jstapleton/synthetic_reads.

## Supporting Information

S1 Fig Detail showing the function of the regions of the barcode adapter during sample preparation. In the multiplexed version of the protocol, the ‘CC’ adjacent to the barcode is replaced by a sample-specific multiplexing index.
(PDF)

S2 Fig Schematic of the steps to convert sheared circular DNA into a sequencing-ready library. Circularized DNA (black) containing barcode and annealing sequences (blue) is fragmented (dotted line) into molecules about 500 bp in length. Some of the resulting molecules contain a barcode and others do not. Asymmetric adapters are ligated to each end of the molecules. Limited-cycle PCR is performed with a first primer complementary to the asymmetric adapter and a second primer complementary to the internal annealing sequence from the tripartite adapter. The primers add the full sequencing adapter sequences to the PCR product. Only molecules containing internal annealing sequences and barcodes are exponentially amplified in the PCR.
(PNG)

S3 Fig Detail showing the function of the regions of the tripartite adapter during sample preparation for two-tube barcode pairing. (PNG)

S4 Fig Overlaid length histograms of synthetic long reads assembled from increasing fractions of the *E*. *coli* MG1655 sequencing data show assembly improvement from barcode pairing. (A) Synthetic reads assembled without barcode pairing. (B) Synthetic reads assembled with barcode pairing. Barcode pairing improves assembly of long synthetic reads, particularly at low coverage (i.e., low fractions of the dataset used).
(PDF)

S5 Fig Barcode pairing improves assembly N50 length. Shown are assembly statistics of synthetic long reads assembled from increasing fractions of the *E*. *coli* MG1655 sequencing data. Blue = without barcode pairing, green = with barcode pairing. (A) The number of synthetic reads longer than 1 kb. Barcode pairing removes duplicate synthetic reads that result from two unpaired barcodes assembling the same or overlapping target fragments. (B) The N50 length of the assembled synthetic reads longer than 1 kb. Barcode pairing increases the N50 length of the assemblies.
(PNG)

S6 Fig (A) Insertion and (B) deletion rates (inserted or deleted nucleotides per aligned position) of synthetic long reads from the *E*. *coli* MG1655 dataset, plotted as a function of relative position. Both distributions indicate indels are most likely in the low-confidence regions near the ends of the assembled synthetic long reads.
(PNG)

S7 Fig GC content distributions of assembled synthetic reads. Grey: *Gelsemium*; blue: potato; green: chicken; red: *E*. *coli* MG1655. Dotted vertical lines indicate the overall GC content of each genome.
(PDF)

S8 Fig (A) Length histogram of synthetic long reads assembled from short reads from a second, independent sample of *G*. *sempervirens* genomic DNA (minimum length 1 kb). The N50 length of the assembly is 2.8 kb. (B) Length histogram of synthetic long reads assembled from *G*. *gallus* genomic reads (minimum length 1 kb). The N50 length of the assembly is 2.2 kb. (C) Length histogram of the synthetic long reads assembled from *S*. *tuberosum* genomic reads (minimum length 1 kb). The N50 length of the assembly is 3.3 kb.
(PNG)

S9 Fig (A) The number of read pairs associated with each barcode in the *G*. *sempervirens* dataset, with a minimum of 50 read pairs. Ideally, the same number of reads would be associated with each barcode. (B) Cumulative probability graph of the read distribution.
(PDF)

S10 Fig Incorporation of a multiplexing index into the barcode-containing adapter allows independently barcoded samples to be mixed and processed in a single tube. Adapter sets containing distinct 6-bp multiplexing indexes (green, orange, yellow, and grey) are ligated to sample DNA in separate, parallel reactions and PCR amplified. The purified, quantified PCR products are mixed, and the intramolecular nature of the key circularization step enables multiplexed library preparation. After sequencing, short reads are demultiplexed according to the 6-bp index sequence that follows the barcode region. A representative forward read is shown. Because the multiplexing index is contained in the forward read, standard Illumina sample multiplexing using a 6- to 8-bp multiplexing read can additionally be used.
(PNG)

S11 Fig Length histograms of twenty-four independent *E*. *coli* genomic samples prepared for sequencing in a single tube using a multiplexed protocol. (PDF)

S12 Fig Schematic diagram of the approach for adding barcodes to full-length cDNA during the reverse-transcription step (Picelli et al. 2013). (1) RNA (purple) is reverse transcribed from a primer consisting of a poly-T annealing region (green) and an overhang containing an Illumina adapter sequence (blue), a barcode (pink stripes), and a PCR primer annealing region (black). The reverse transcriptase adds several non-templated dC bases to the 3’ end of the newly synthesized strand. (2) dG bases at the 3’ end of a template-switching oligonucleotide (TSO) anneal to the overhanging non-templated dC bases. The TSO consists of a PCR annealing region (black), a second barcode region (green hashed), a second Illumina adapter sequence (red), and the 3’ dG bases. (3) The reverse transcriptase template-switches to copy the TSO and further extend the 3’ end of the first DNA strand. (4) The second strand is synthesized and full-length cDNA is exponentially amplified by PCR with a single primer (black).
(PNG)

S13 Fig Version of Fig 2C with a standard axis. Box plots showing the number of splice junctions spanned by short reads and synthetic long reads.
(PDF)

S14 Fig Visualization of alignments shows examples of junctions where splicing differs between HCT116 and HepG2 cell lines (A, B, C), and a novel transcript in the HCT116 cell line (D). The aligned long reads are shown in IGV (Integrative Genomics Viewer). (A) Synthetic reads indicate novel alternative 5' splice sites of two exons on gene GPX4, which are differentially spliced in the HCT116 and HepG2 cell lines. (B) Novel variable 5' splice sites on gene PPP4C expressed in the HepG2 cell line. (C) A novel exon on gene PSAP expressed in the HCT116 cell line. (D) Assembled long reads identify a novel transcript on chromosome 6 expressed in the HCT116 cell line.
(EPS)

S15 Fig 3D scatter plot showing barcode fidelity in sequencing results from a mixture of six plasmids. The reads associated with each barcode were searched for short sequences unique to each variant. Each point represents a different barcode (8,108 total) and its position indicates the number of times sequences unique to each of three of the mixed target molecules were found within that set of barcode-grouped reads. Counting the barcodes associated with each target provides a measurement of mixture composition. Note that although Target 3 is rare in the mixture, the barcodes that tag it have as many counts as barcodes tagging more abundant targets.
(PDF)

S16 Fig Detail showing the function of the regions of the tripartite adapter during sample preparation for one-tube barcode pairing. (PDF)

S1 Note Alternative barcode pairing protocols. (DOCX)

S1 Table Synthetic long read assembly statistics. (DOCX)

S2 Table Accuracy of synthetic long reads aligned against the MG1655 genome. (DOCX)

S3 Table *S*. *tuberosum* synthetic read alignment statistics. (DOCX)

S4 Table Summary of synthetic reads used in the synthetic read scaffolded *G*. *sempervirens* assembly. (DOCX)

S5 Table Evaluation of the *G*. *sempervirens* assemblies using the CEGMA pipeline. (DOCX)

S6 Table Alignment of RNA-seq reads to the *G*. *sempervirens* assemblies. (DOCX)

S7 Table Multiplexed synthetic long read assembly statistics. (DOCX)

S8 Table Comparison of synthetic long read approaches for genome assembly and phasing. (DOCX)

S9 Table Human mRNA splice variant analysis. (DOCX)

S10 Table Best-supported HCT116 mRNA synthetic reads spanning novel splice junctions. (DOCX)

S11 Table Best-supported HepG2 mRNA synthetic reads spanning novel splice junctions. (DOCX)

S12 Table Comparison of application categories enabled by different synthetic long read methods. (DOCX)

S13 Table Oligonucleotides used in library preparation. (DOCX)

S14 Table Barcode adapter oligonucleotides (Oligo 1 in S13 Table) for multiplexed library preparation. (DOCX)
