# Supplementary material for: Haplotype-Phased Synthetic Long Reads from Short-Read Sequencing
Source: PLoS One. 2016 Jan 20;11(1):e0147229. doi: 10.1371/journal.pone.0147229 (PMC4720449; doi:10.1371/journal.pone.0147229)
Supplement: S1 Fig — In the multiplexed version of the protocol, the ‘CC’ adjacent to the barcode is replaced by a sample-specific multiplexing index. (PDF) [file pone.0147229.s001.pdf]

Sample Preparation: (only one strand is shown, in the 5’ to 3’ direction)

Tripartite adapter is ligated to the end of the target molecule:
Ligated target – NNN CCTACACGACGCTCTTCCGATCT NNNNNNNNNNNNNNNN CC AGGAATAGTTATGTGCATTAATGAATGG CGCC

↓

Target molecules with adapters at both ends are amplified and the PCR primer annealing region is removed:
Ligated target – NNN CCTACACGACGCTCTTCCGATCT NNNNNNNNNNNNNNNN CC

↓

Amplified target molecules are fragmented and circularized:
Ligated target end – NNN CCTACACGACGCTCTTCCGATCT NNNNNNNNNNNNNNNN CC – ligated region of interest

↓

Circularized DNA is fragmented and fragments containing adapter sequences are prepared for sequencing:
Illumina adapter 1 – CCTACACGACGCTCTTCCGATCT NNNNNNNNNNNNNNNN CC – ligated region of interest – Illumina adapter 2

↓

Resulting sequencing read:
NNNNNNNNNNNNNNNN CC – ligated region of interest

↓

In computational pipeline, the sequences at the start of the read are used to determine the sample and target molecule of origin:
NNNNNNNNNNNNNNNN CC – ligated region of interest

↓	↓	↓
Determines target molecule of origin	Confirms upstream sequence is a barcode	Contains sequence information
