# Supplementary figures and images for: Haplotype-Phased Synthetic Long Reads from Short-Read Sequencing
Source: PLoS One. 2016 Jan 20;11(1):e0147229. doi: 10.1371/journal.pone.0147229 (PMC4720449; doi:10.1371/journal.pone.0147229)

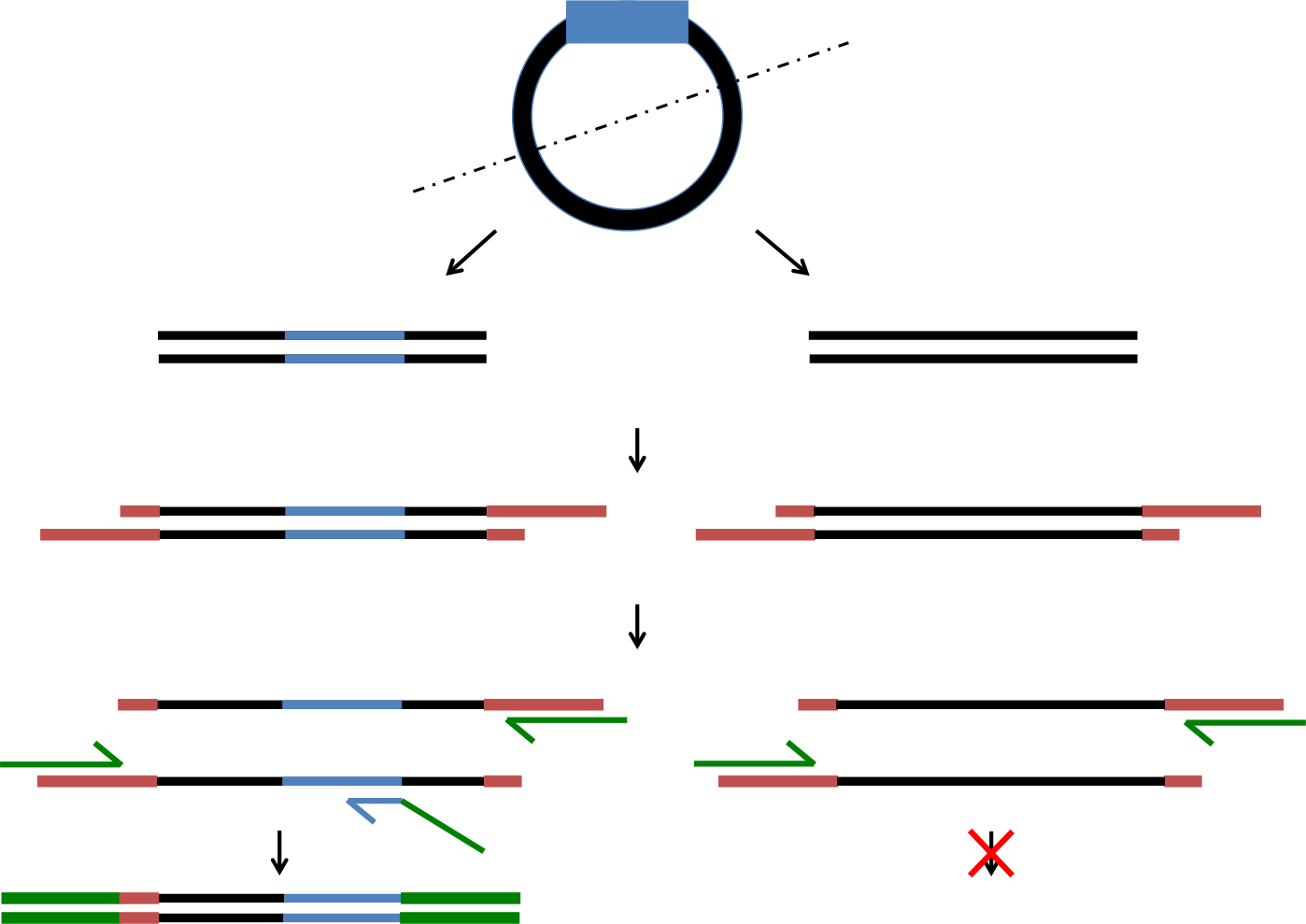

Supplement: S2 Fig — Circularized DNA (black) containing barcode and annealing sequences (blue) is fragmented (dotted line) into molecules about 500 bp in length. Some of the resulting molecules contain a barcode and others do not. Asymmetric adapters are ligated to each end of the molecules. Limited-cycle PCR is performed with a first primer complementary to the asymmetric adapter and a second primer complementary to the internal annealing sequence from the tripartite adapter. The primers add the full sequencing adapter sequences to the PCR product. Only molecules containing internal annealing sequences and barcodes are exponentially amplified in the PCR. (PNG) [file pone.0147229.s002.png]

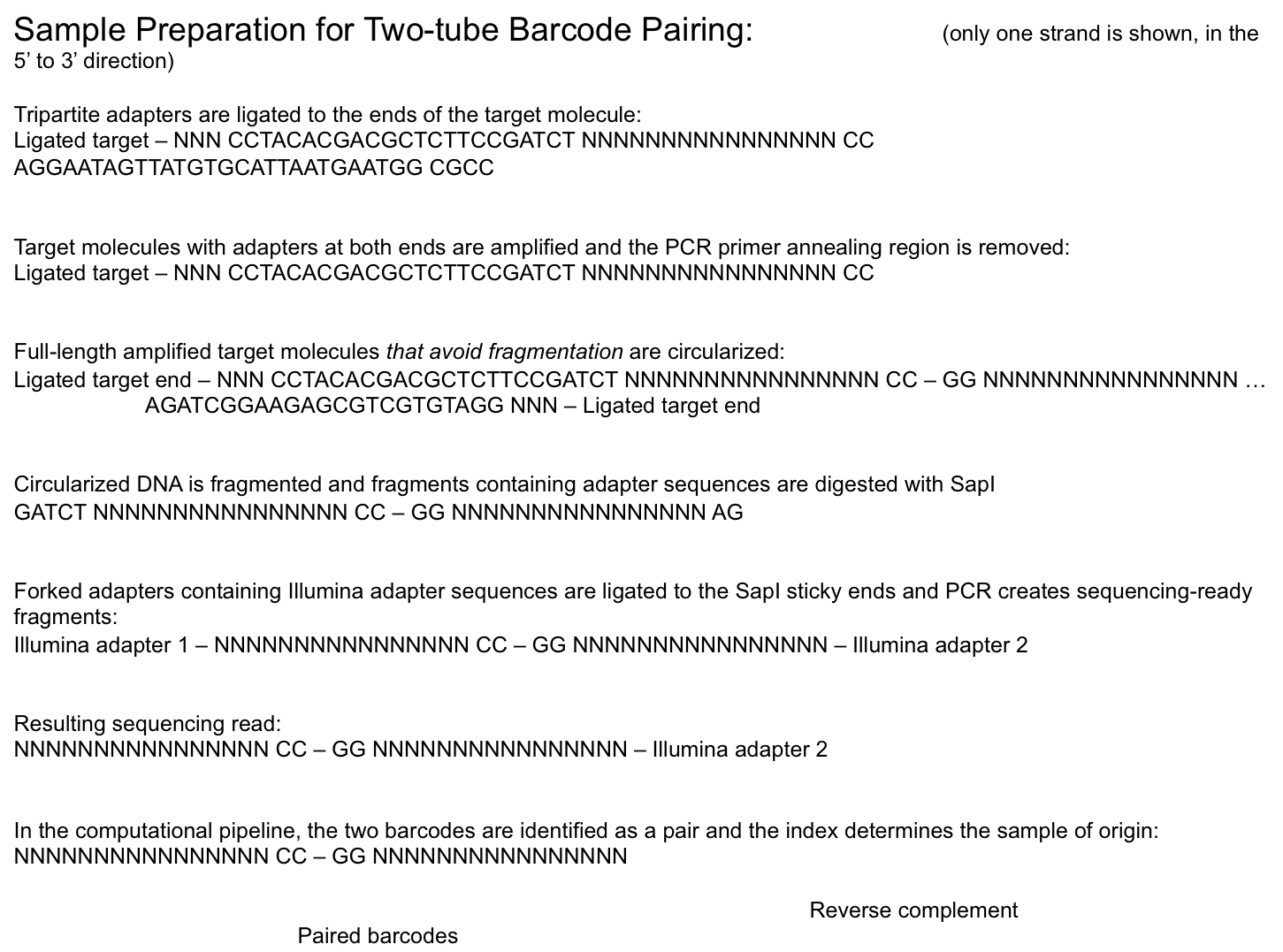

Supplement: S3 Fig — (PNG) [file pone.0147229.s003.png]

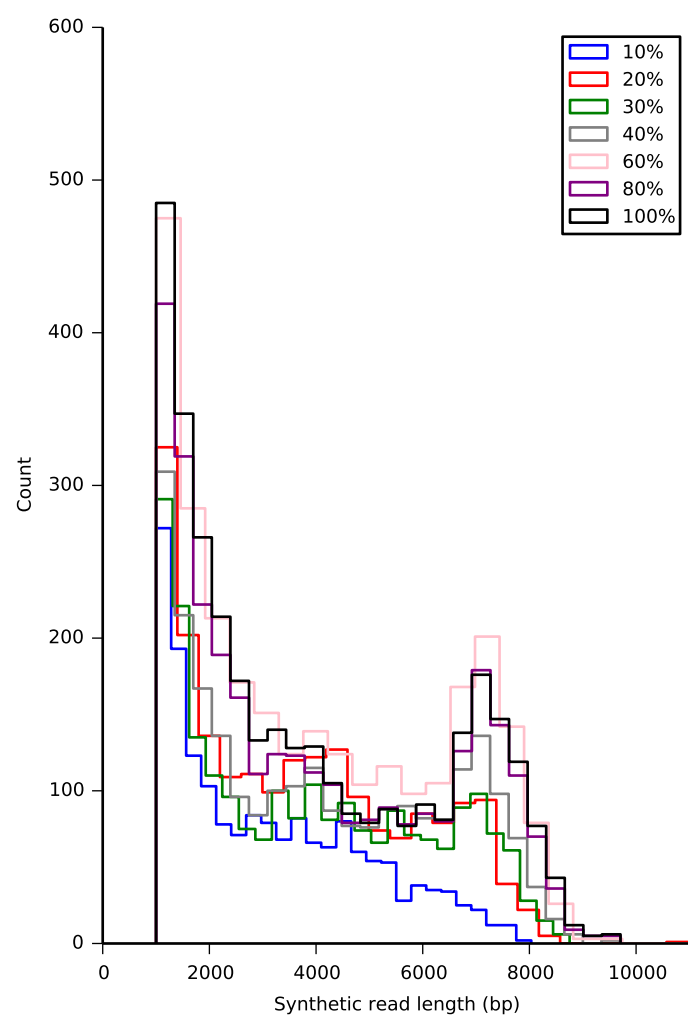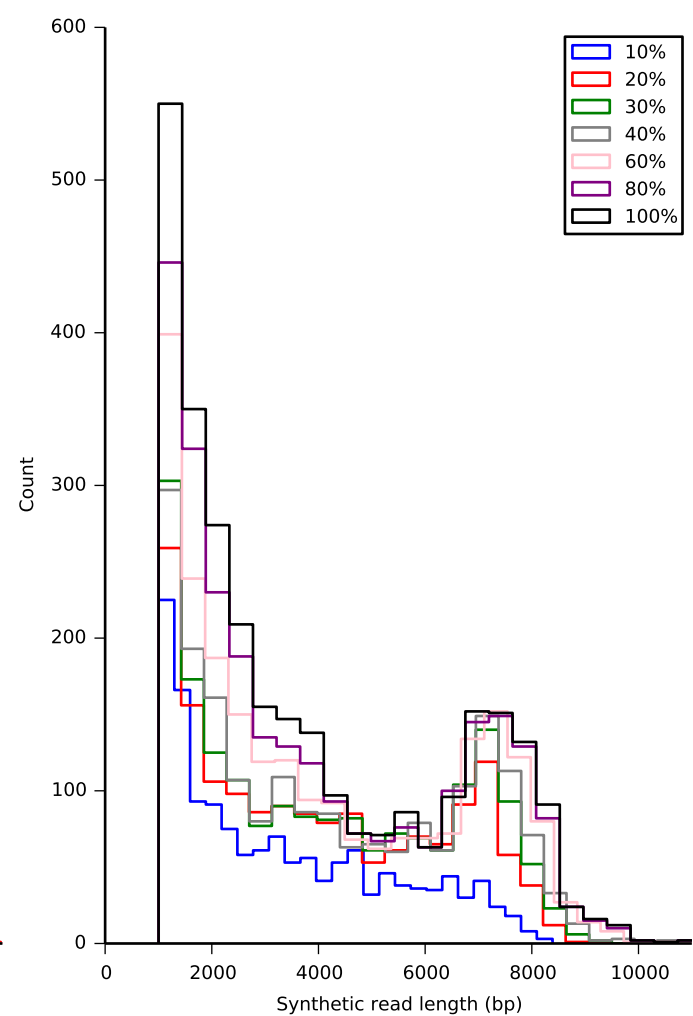

Supplement: S4 Fig — (A) Synthetic reads assembled without barcode pairing. (B) Synthetic reads assembled with barcode pairing. Barcode pairing improves assembly of long synthetic reads, particularly at low coverage (i.e., low fractions of the dataset used). (PDF) [file pone.0147229.s004.pdf]

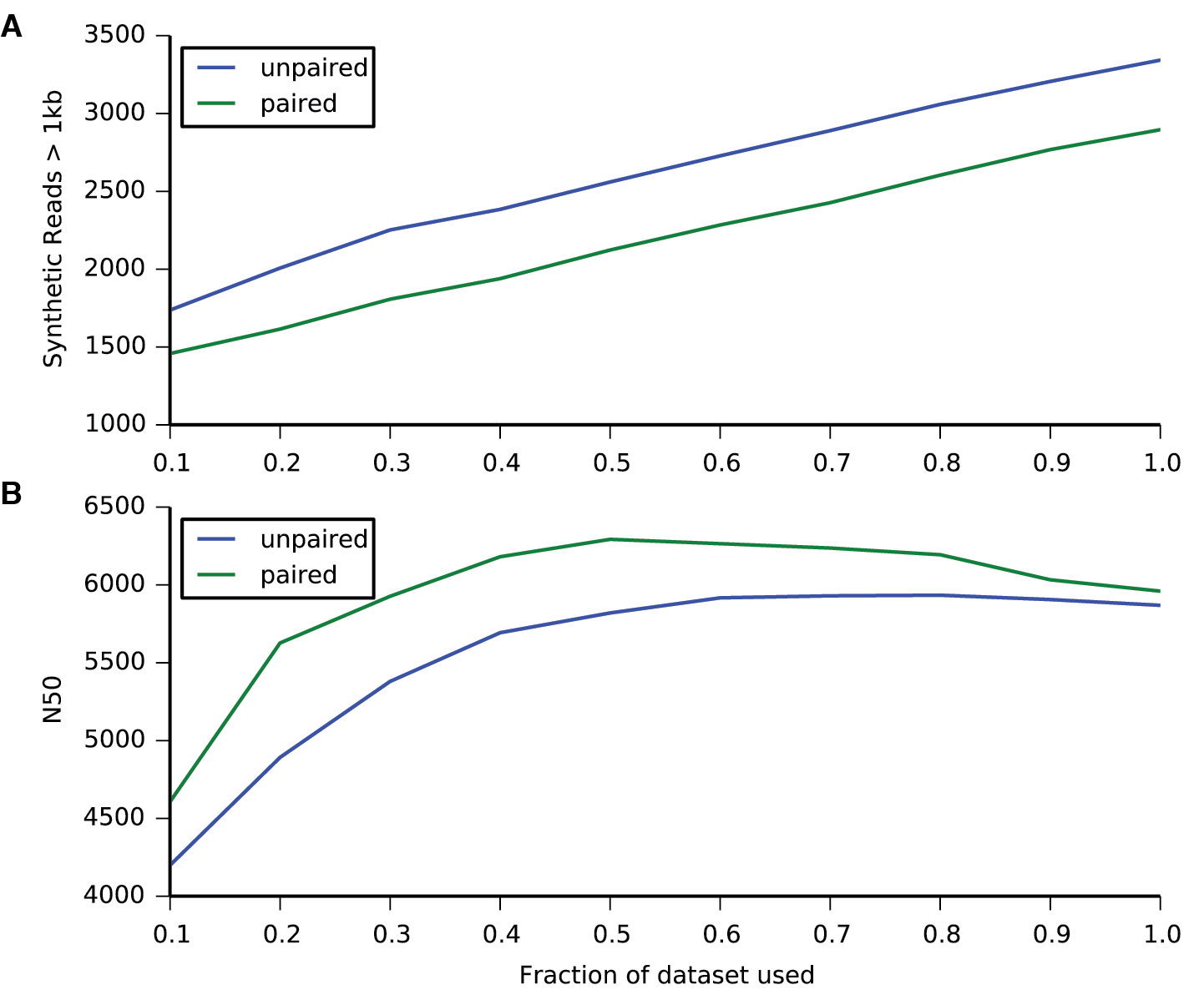

Supplement: S5 Fig — Shown are assembly statistics of synthetic long reads assembled from increasing fractions of the E. coli MG1655 sequencing data. Blue = without barcode pairing, green = with barcode pairing. (A) The number of synthetic reads longer than 1 kb. Barcode pairing removes duplicate synthetic reads that result from two unpaired barcodes assembling the same or overlapping target fragments. (B) The N50 length of the assembled synthetic reads longer than 1 kb. Barcode pairing increases the N50 length of the assemblies. (PNG) [file pone.0147229.s005.png]

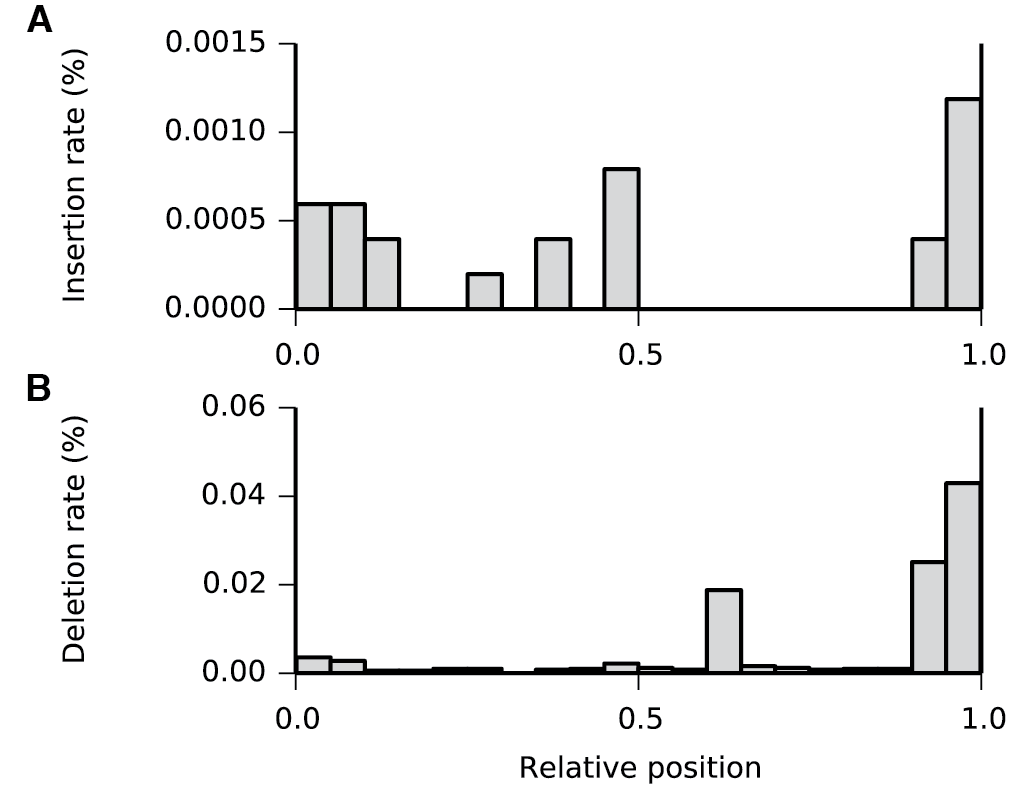

Supplement: S6 Fig — Both distributions indicate indels are most likely in the low-confidence regions near the ends of the assembled synthetic long reads. (PNG) [file pone.0147229.s006.png]

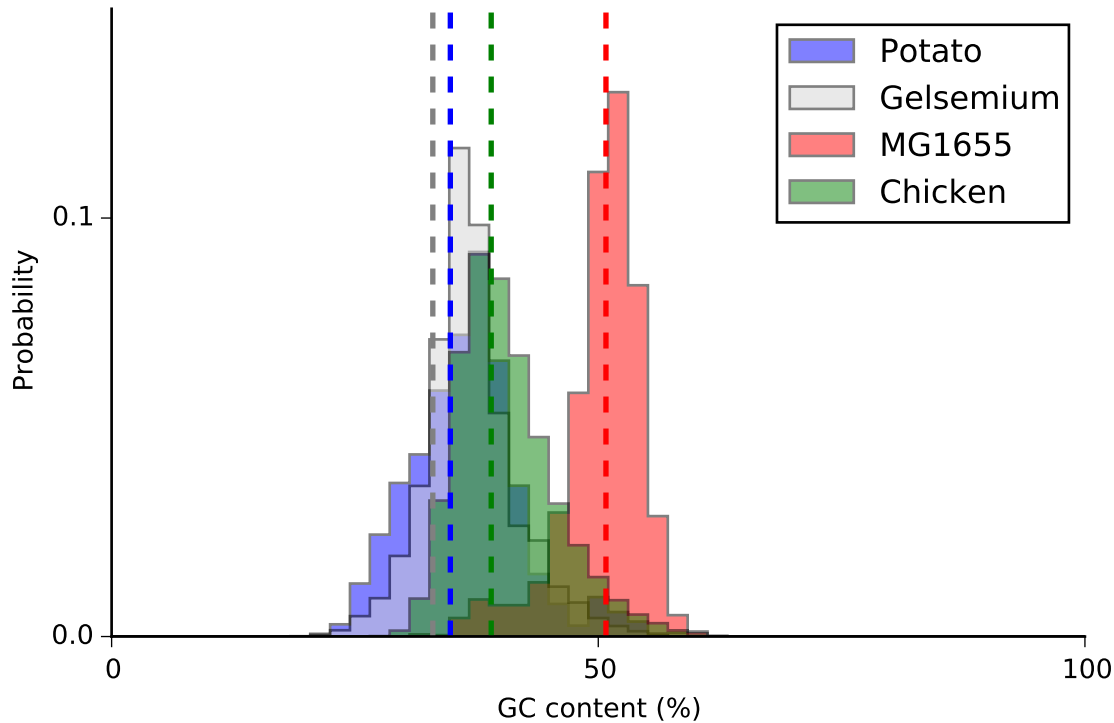

Supplement: S7 Fig — Grey: Gelsemium; blue: potato; green: chicken; red: E. coli MG1655. Dotted vertical lines indicate the overall GC content of each genome. (PDF) [file pone.0147229.s007.pdf]

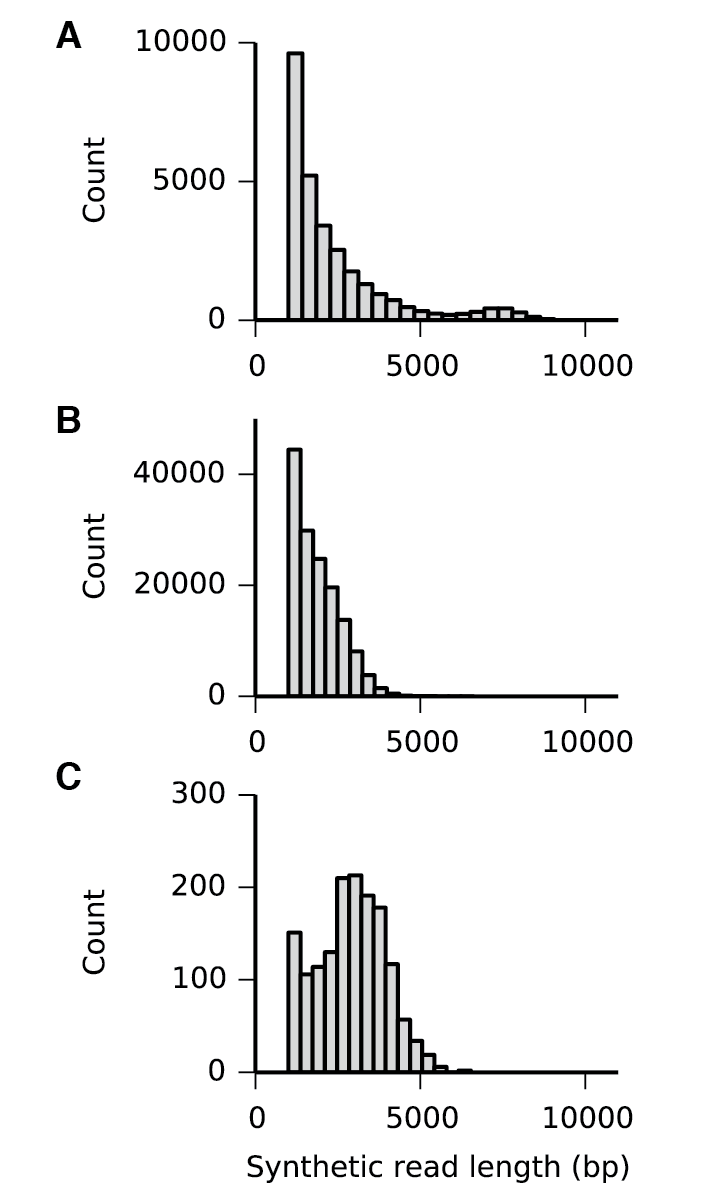

Supplement: S8 Fig — (A) Length histogram of synthetic long reads assembled from short reads from a second, independent sample of G. sempervirens genomic DNA (minimum length 1 kb). The N50 length of the assembly is 2.8 kb. (B) Length histogram of synthetic long reads assembled from G. gallus genomic reads (minimum length 1 kb). The N50 length of the assembly is 2.2 kb. (C) Length histogram of the synthetic long reads assembled from S. tuberosum genomic reads (minimum length 1 kb). The N50 length of the assembly is 3.3 kb. (PNG) [file pone.0147229.s008.png]

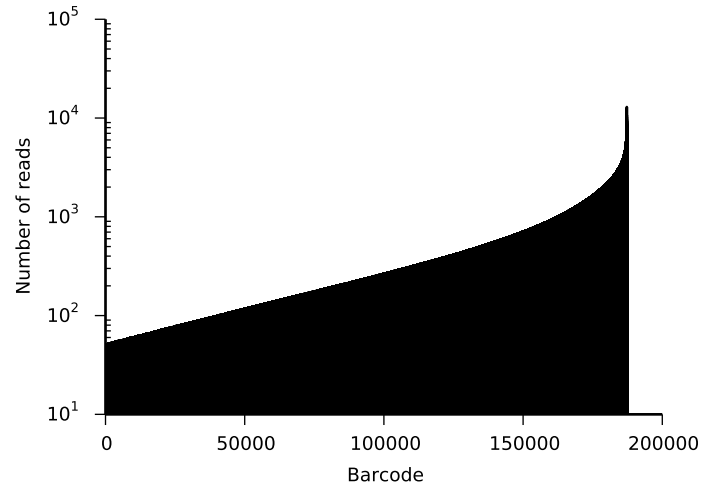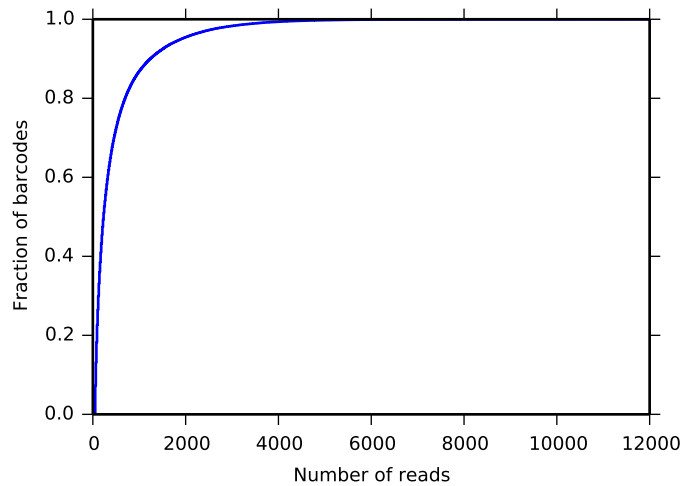

Supplement: S9 Fig — (A) The number of read pairs associated with each barcode in the G. sempervirens dataset, with a minimum of 50 read pairs. Ideally, the same number of reads would be associated with each barcode. (B) Cumulative probability graph of the read distribution. (PDF) [file pone.0147229.s009.pdf]

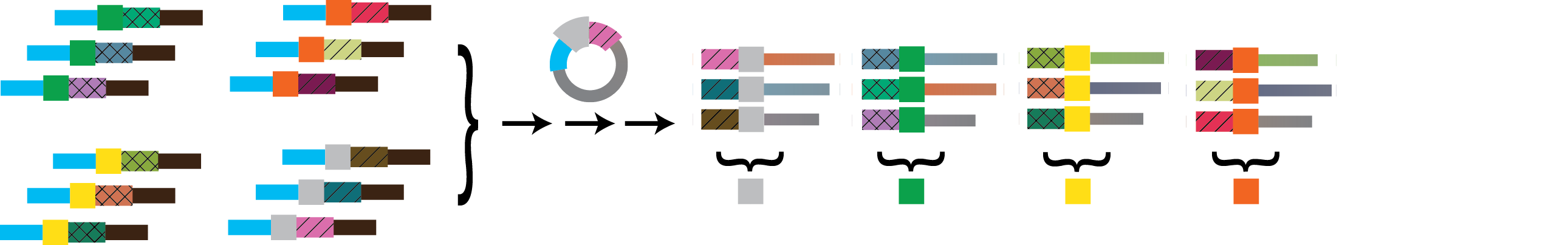

Supplement: S10 Fig — Adapter sets containing distinct 6-bp multiplexing indexes (green, orange, yellow, and grey) are ligated to sample DNA in separate, parallel reactions and PCR amplified. The purified, quantified PCR products are mixed, and the intramolecular nature of the key circularization step enables multiplexed library preparation. After sequencing, short reads are demultiplexed according to the 6-bp index sequence that follows the barcode region. A representative forward read is shown. Because the multiplexing index is contained in the forward read, standard Illumina sample multiplexing using a 6- to 8-bp multiplexing read can additionally be used. (PNG) [file pone.0147229.s010.png]

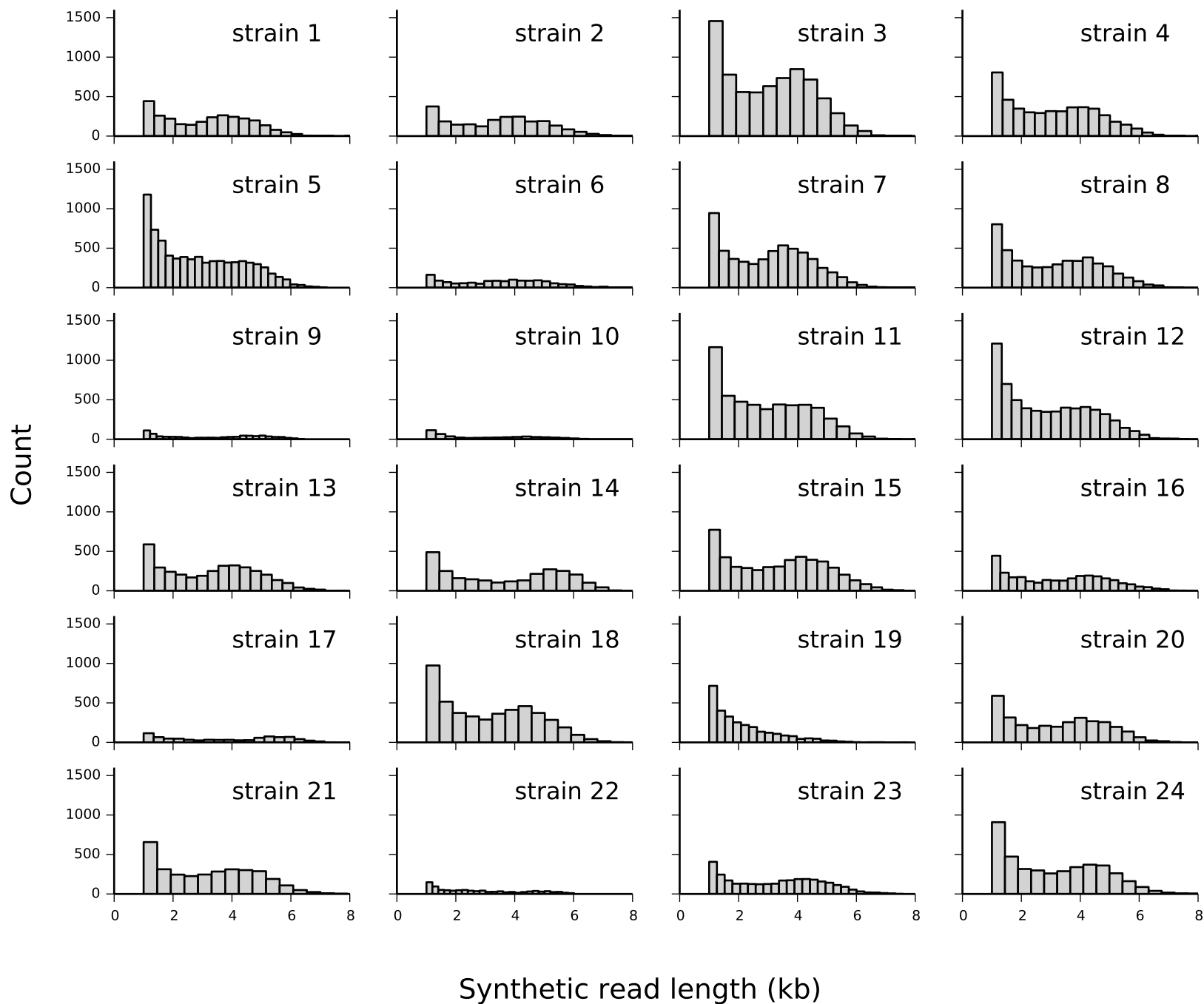

Supplement: S11 Fig — (PDF) [file pone.0147229.s011.pdf]

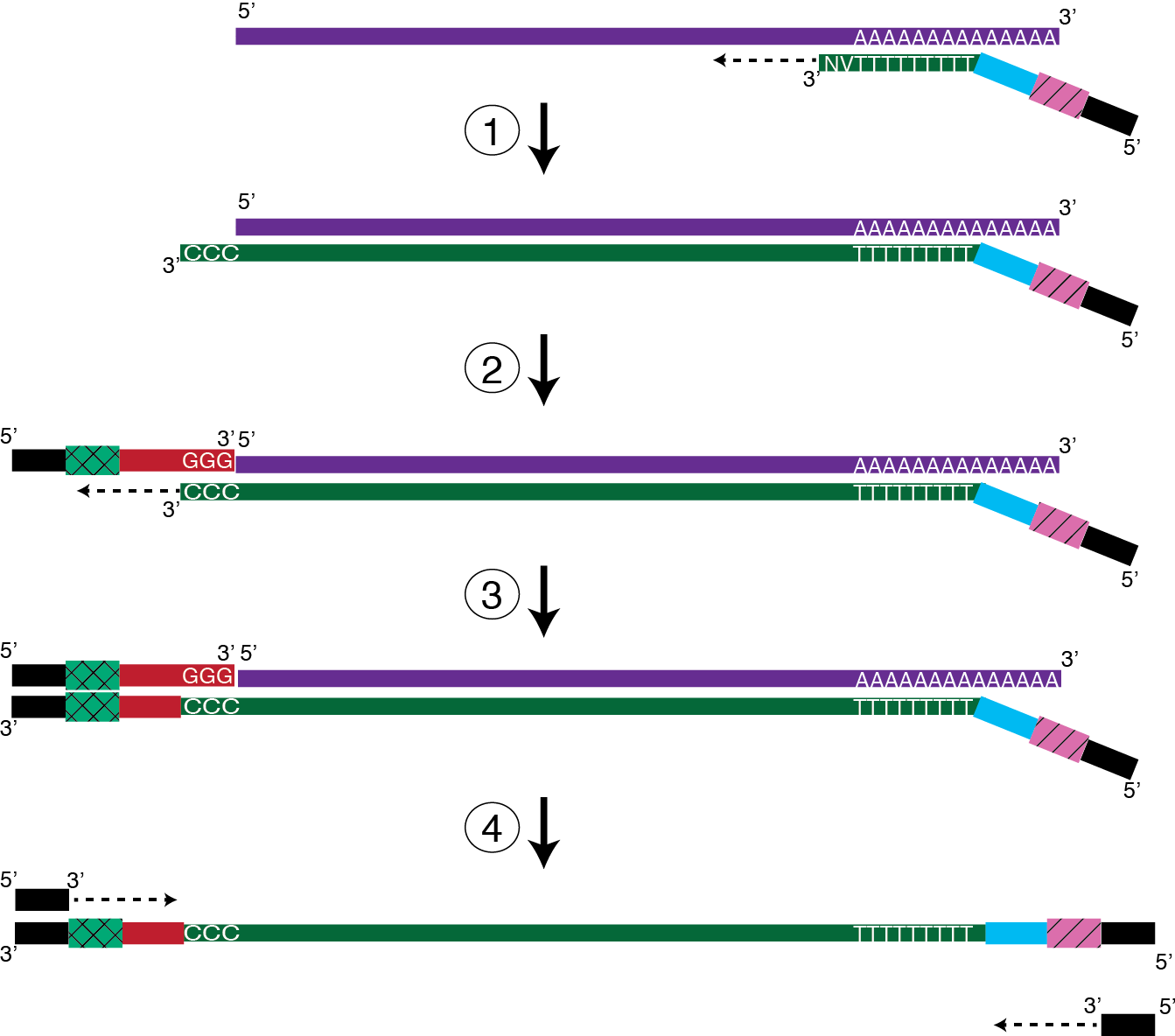

Supplement: S12 Fig — (1) RNA (purple) is reverse transcribed from a primer consisting of a poly-T annealing region (green) and an overhang containing an Illumina adapter sequence (blue), a barcode (pink stripes), and a PCR primer annealing region (black). The reverse transcriptase adds several non-templated dC bases to the 3’ end of the newly synthesized strand. (2) dG bases at the 3’ end of a template-switching oligonucleotide (TSO) anneal to the overhanging non-templated dC bases. The TSO consists of a PCR annealing region (black), a second barcode region (green hashed), a second Illumina adapter sequence (red), and the 3’ dG bases. (3) The reverse transcriptase template-switches to copy the TSO and further extend the 3’ end of the first DNA strand. (4) The second strand is synthesized and full-length cDNA is exponentially amplified by PCR with a single primer (black). (PNG) [file pone.0147229.s012.png]

Junctions spanned

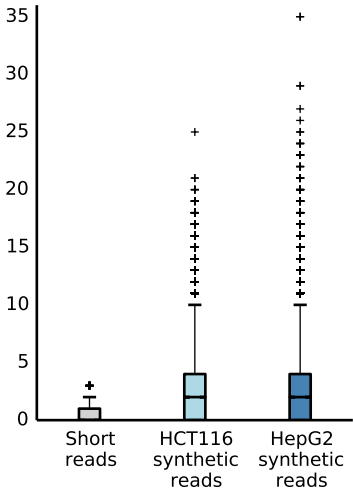

Supplement: S13 Fig — Box plots showing the number of splice junctions spanned by short reads and synthetic long reads. (PDF) [file pone.0147229.s013.pdf]

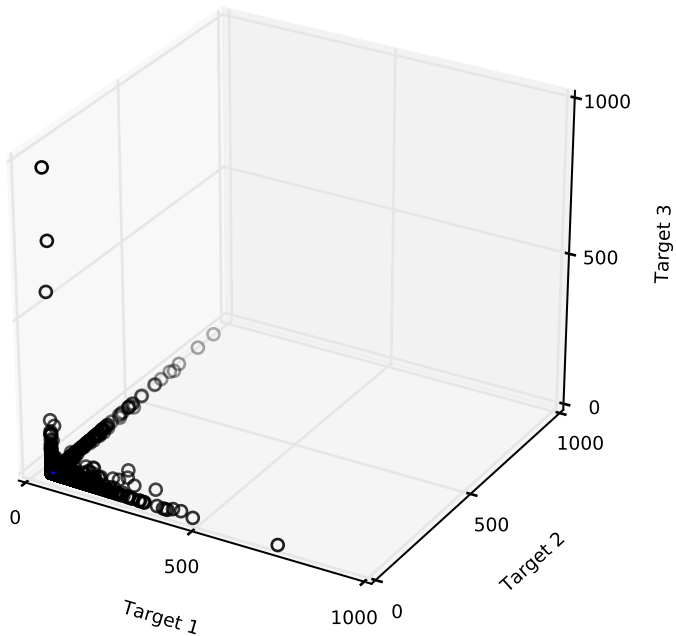

Supplement: S15 Fig — The reads associated with each barcode were searched for short sequences unique to each variant. Each point represents a different barcode (8,108 total) and its position indicates the number of times sequences unique to each of three of the mixed target molecules were found within that set of barcode-grouped reads. Counting the barcodes associated with each target provides a measurement of mixture composition. Note that although Target 3 is rare in the mixture, the barcodes that tag it have as many counts as barcodes tagging more abundant targets. (PDF) [file pone.0147229.s015.pdf]

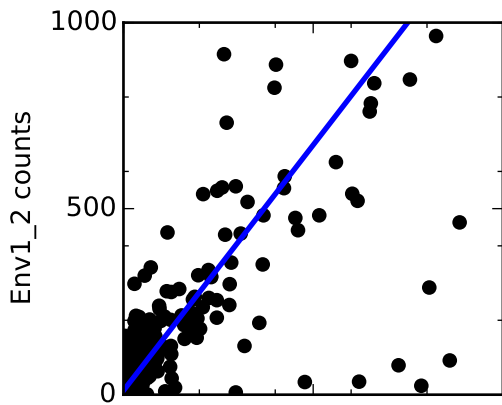

$\text{Env1\_2} = 11.06 + 1.32 \cdot \text{Env1\_1}$   
P-value < 0.000001  
 $R^2 = 0.887$

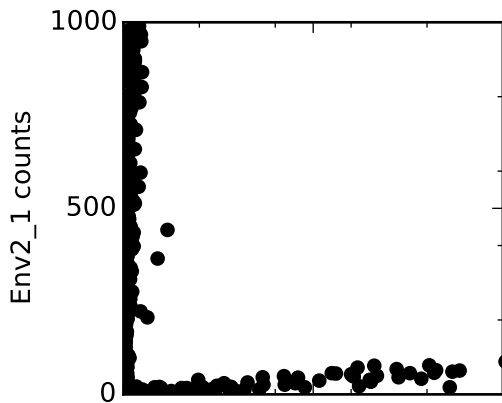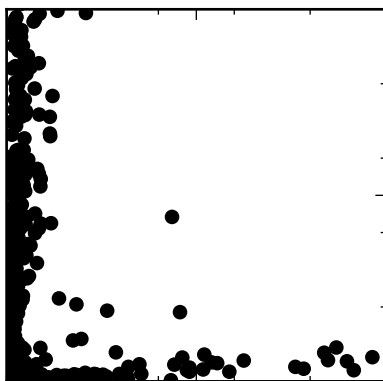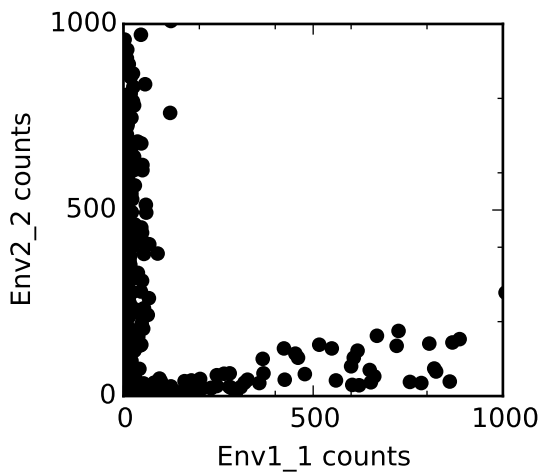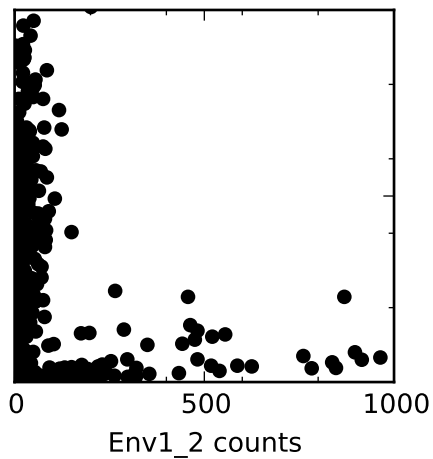

$\text{Env2\_2} = 152.23 + 1.24 \cdot \text{Env2\_1}$   
P-value < 0.000001  
 $R^2 = 0.312$

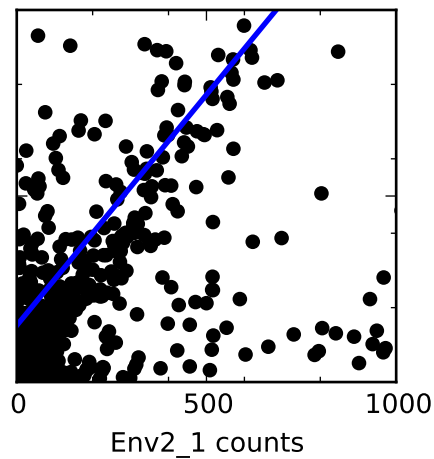

Supplement: S16 Fig — (PDF) [file pone.0147229.s016.pdf]
