# Supplementary material for: Haplotype-Phased Synthetic Long Reads from Short-Read Sequencing
Source: PLoS One. 2016 Jan 20;11(1):e0147229. doi: 10.1371/journal.pone.0147229 (PMC4720449; doi:10.1371/journal.pone.0147229)
Supplement: S1 Note — (DOCX) [file pone.0147229.s017.docx]

**S1 Note. Alternative barcode pairing protocols.** Two separate protocols were developed for barcode pairing, the two-tube protocol and the one-tube protocol. The one-tube protocol was used for the MG1655 experiment, and the two-tube protocol was used for the multiplexed *E. coli*, mRNA, and *env* experiments. Barcode pairing was not used in the *G. gallus*, *S. tuberosum*, or *G. sempervirens* experiments.

The one-tube protocol (Supplementary Fig. 16) has the advantage of sample preparation occurring entirely in a single tube. However, only a fraction of the total barcodes can be paired (1/2 when two adapters are used). A mixture of two or more barcode-containing adapters is ligated to the dT-tailed target fragments. The adapters differ in their sequencing primer region. We used sequences derived from the Illumina Universal and Index primer sequences, respectively. As a result, approximately half of the target fragments will have different sequencing regions in the adapters that ligate to the two ends. Following PCR, some fraction of the full-length copies will avoid fragmentation, and circularization will bring the two barcodes together. Downstream limited-cycle PCR (lcPCR) will fail to amplify molecules that have the same adapter at each end because the identical sequencing regions outside the barcode regions will form a tight hairpin upon becoming single stranded. However, in molecules with different adapters at the ends, no hairpin will form, and addition of a primer complementary to the second sequencing region enables amplification of the paired barcodes. In the computational pipeline, paired-barcode reads are identified, trimmed of adapter sequences, and parsed to extract the barcode pairs.

The two-tube protocol (Supplementary Fig. 3**)** adds the complexity of splitting the library preparation into two tubes for the last third of the protocol, one tube to generate barcoded target reads and a second solely to generate paired barcode reads. The advantage is improved control of the fraction of the eventual short reads of each type. In this protocol, only one adapter sequence is used, so all target molecules ligate the same adapter at both ends. As a result, all molecules derived from circularized full-length amplicons will form a tight hairpin during lcPCR, and no paired-barcode reads will be present in the main sequencing sample. Following attachment to streptavidin-coated beads and prior to ligation of asymmetric adapters, a fraction (~15%) of the beads are moved to a second tube. SapI digestion cuts a site in the sequencing region (taken from the Illumina Multiplexing Sample Prep Oligo Only Kit), leaving sticky ends. Y-shaped adapters are ligated to the sticky ends to provide PCR annealing regions, and subsequent lcPCR adds the requisite sequencing adapter regions and a multiplexing index that allows barcode-pairing reads to be identified during analysis.
