# Supplementary material for: Haplotype-Phased Synthetic Long Reads from Short-Read Sequencing
Source: PLoS One. 2016 Jan 20;11(1):e0147229. doi: 10.1371/journal.pone.0147229 (PMC4720449; doi:10.1371/journal.pone.0147229)
Supplement: S1 Table — (DOCX) [file pone.0147229.s018.docx]

| **S1 Table.** Synthetic long read assembly statistics. | | | | |  |
| --- | --- | --- | --- | --- | --- |
| Sample | Trimmed, filtered 2x150 bp read pairs | Synthetic reads > 1 kb | N50 length (kb) | Short-read bases per synthetic read base^a^ | |
| *E. coli* MG1655 | 8,124,591 | 2,878 | 6.0 | 221.4 | |
| *G. sempervirens* | 112,289,622 | 149,447^b^ | 3.9 | 75.8 | |
| *G. sempervirens* #2 | 10,019,885 | 28,574 | 2.8 | 43.6 | |
| *G. gallus* (chicken) | 103,601,271 | 125,203 | 2.0 | 113.3 | |
| *S. tuberosum* (potato)^c^ | 2,789,741 | 1,528 | 3.3 | 188.9 | |
| Recombinant *E. coli* from evolution experiment (sum of 24 strains) | 201,717,764 | 87,395 | 4.0 | 224.7 | |
| HCT116 mRNA | 85,118,973 | 11,707 | 1.5 | N/A | |
| HepG2 mRNA | 43,827,058 | 6,640 | 1.6 | N/A | |
| HIV *env* mixture | 51,127,680 | 7,723^d^ | 2.3 | N/A | |

^a^Calculated as (total short-read nucleotides) / (total nucleotides in synthetic reads > 1 kb). Not calculated for mRNA samples, where many synthetic reads were shorter than 1 kb due to RNA degradation and the natural length distribution of mammalian mRNA, or for the HIV sample, which was deliberately over-sequenced. Low-quality or adapter-sequence nucleotides trimmed from short reads were included, i.e., the numerator was (2*150*number of read pairs).

^b^Of these,111,054 synthetic reads longer than 1.5 kb with an N50 of 4.3 kb were used to scaffold the draft genome.

^c^100 bp were trimmed from the ends of these synthetic reads prior to alignment, yielding 1,411 reads > 1 kb with an N50 length of 3.1 kb.

^d^Additional steps to remove duplicate synthetic reads reduced this number to 1,173.
