# Supplementary material for: Haplotype-Phased Synthetic Long Reads from Short-Read Sequencing
Source: PLoS One. 2016 Jan 20;11(1):e0147229. doi: 10.1371/journal.pone.0147229 (PMC4720449; doi:10.1371/journal.pone.0147229)
Supplement: S2 Table — (DOCX) [file pone.0147229.s019.docx]

| **S2 Table.** Accuracy of synthetic long reads aligned against the MG1655 genome. | |
| --- | --- |
| Reference genome | Aligned bases |
| Aligned bases | 10,162,249 |
| Mismatches | 3,897 |
| Mismatch rate | 0.00038 |
| Insertions | 23 |
| Insertion rate | 2.263e-6 |
| Deletions | 545 |
| Deletion rate | 5.363e-5 |
| Clipped bases | 1,205,861 |
| Percent clipped (hard + soft) | 10.61 |
| A 🡪 C | 2.3% |
| A 🡪 G | 25.1% |
| A🡪 T | 6.3% |
| C 🡪 A | 2.7% |
| C 🡪 G | 1.5% |
| C 🡪 T | 10.9% |
| G 🡪 A | 10.5% |
| G 🡪 C | 1.3% |
| G 🡪 T | 2.7% |
| T 🡪 A | 5.3% |
| T 🡪 C | 29.2% |
| T 🡪 G | 1.9% |
