# Supplementary material for: Haplotype-Phased Synthetic Long Reads from Short-Read Sequencing
Source: PLoS One. 2016 Jan 20;11(1):e0147229. doi: 10.1371/journal.pone.0147229 (PMC4720449; doi:10.1371/journal.pone.0147229)
Supplement: S3 Table — (DOCX) [file pone.0147229.s020.docx]

**S3 Table.** *S. tuberosum* synthetic read alignment statistics.

| Mapping Quality (MapQ) cutoff | Total alignments | % Total synthetic reads |
| --- | --- | --- |
| Unaligned | 38 | 2.7% |
| Multimapping (MapQ = 0) | 11 | 0.8% |
| MapQ >= 30 | 1,329 | 94.2% |
| MapQ >= 60 | 1,291 | 91.5% |

1,411 synthetic reads were aligned to the potato draft genome (v 4.03) with BWA-MEM.
