# Supplementary material for: Haplotype-Phased Synthetic Long Reads from Short-Read Sequencing
Source: PLoS One. 2016 Jan 20;11(1):e0147229. doi: 10.1371/journal.pone.0147229 (PMC4720449; doi:10.1371/journal.pone.0147229)
Supplement: S4 Table — (DOCX) [file pone.0147229.s021.docx]

**S4 Table.** Summary of synthetic reads used in the synthetic read scaffolded *G. sempervirens* assembly.

| Feature | Metric |
| --- | --- |
| Number of Reads | 111,054 |
| Total Read Size | 397.8 Mb |
| Estimated Genome Coverage | 1.3x |
| Maximum Read Length | 15,260 bp |
| Minimum Read Length | 1,500 bp |
| Average Read Length | 3,581 bp |
| Median Read Length | 3,257 bp |
