# Supplementary material for: Haplotype-Phased Synthetic Long Reads from Short-Read Sequencing
Source: PLoS One. 2016 Jan 20;11(1):e0147229. doi: 10.1371/journal.pone.0147229 (PMC4720449; doi:10.1371/journal.pone.0147229)
Supplement: S5 Table — (DOCX) [file pone.0147229.s022.docx]

| **S5 Table.** Evaluation of the *G. sempervirens* assemblies using the CEGMA pipeline. | | |
| --- | --- | --- |
|  | Shotgun contigs | Synthetic read scaffolds |
| Complete (no, %) | 201 (81.05%) | 203 (81.85%) |
| Partial (no, %) | 239 (96.37%) | 241 (97.18%) |
| Genome assembly quality was assessed using the CEGMA pipeline pipeline (Parra et al. 2007), which identifies 248 highly conserved eukaryotic genes in an assembly. Target genes identified with > 70% coverage are indicated as complete, while those identified with less than 70% coverage are indicated as partial. The number of genes identified from each genome assembly is shown, with the percentage of the total 248 in parentheses. | | |
