# Supplementary material for: Haplotype-Phased Synthetic Long Reads from Short-Read Sequencing
Source: PLoS One. 2016 Jan 20;11(1):e0147229. doi: 10.1371/journal.pone.0147229 (PMC4720449; doi:10.1371/journal.pone.0147229)
Supplement: S6 Table — (DOCX) [file pone.0147229.s023.docx]

| **S6 Table.** Alignment of RNA-seq reads to the *G. sempervirens* assemblies. | | | | | |
| --- | --- | --- | --- | --- | --- |
|  |  | Shotgun contigs | | Synthetic read scaffolds | |
| Tissue | Total no. of cleaned reads | No. of reads aligned in multiple mapping (%) | No. of reads aligned in single mapping (%) | No. of reads aligned in multiple mapping (%) | No. of reads aligned in single mapping (%) |
| Immature leaf | 48,752,558 | 40,298,622 (82.66) | 38,865,127 (79.72) | 40,442,197 (82.95) | 38,893,695 (79.78) |
| Stem | 77,935,967 | 54,580,534 (70.03) | 52,047,211 (66.78) | 55,007,503 (70.58) | 52,284,194 (67.09) |
| Stamens | 61,775,435 | 48,801,351 (79.00) | 46,386,521 (75.09) | 48,964,224 (79.26) | 46,376,610 (75.07) |
| Pistils | 43,707,006 | 35,017,604 (80.12) | 33,605,249 (76.89) | 35,118,309 (80.35) | 33,644,121 (76.98) |
| Petal | 57,708,901 | 44,982,737 (77.95) | 42,838,719 (74.23) | 45,133,271 (78.21) | 42,915,754 (74.37) |
| Cleaned RNA-seq reads from five tissues were aligned to shotgun contigs and synthetic read scaffolds. For each assembly, the number of reads that aligned to genome in multiple and single mapping mode is shown with the percentage of reads in parentheses. | | | | | |
|  |  |  |  |  |  |
|  |  |  |  |  |  |
