# Supplementary material for: Haplotype-Phased Synthetic Long Reads from Short-Read Sequencing
Source: PLoS One. 2016 Jan 20;11(1):e0147229. doi: 10.1371/journal.pone.0147229 (PMC4720449; doi:10.1371/journal.pone.0147229)
Supplement: S7 Table — (DOCX) [file pone.0147229.s024.docx]

| **S7 Table.** Multiplexed synthetic long read assembly statistics. | | | | |
| --- | --- | --- | --- | --- |
| *E. coli* strain number | Trimmed, filtered 2x150 bp read pairs | Contigs >1kb | N50 length (kb) | *E. coli* genome coverage |
| Overall combined | 201,717,764 | 87,395 | 4.006 | 2.44 |
| REL11734 | 8,872,508 | 2,868 | 3.921 | 1.94 |
| REL11735 | 11,930,637 | 2,366 | 4.218 | 1.74 |
| REL11736 | 10,809,493 | 7,262 | 3.866 | 4.77 |
| REL11737 | 7,954,228 | 4,673 | 3.914 | 3.09 |
| REL11738 | 10,911,001 | 7,782 | 3.746 | 4.86 |
| REL11739 | 6,665,185 | 1,454 | 4.319 | 1.10 |
| REL11740 | 13,681,197 | 5,766 | 3.749 | 3.78 |
| REL11741 | 8,853,848 | 4,806 | 3.976 | 3.21 |
| REL11742 | 9,302,047 | 800 | 4.487 | 0.57 |
| REL11743 | 7,254,760 | 508 | 4.204 | 0.33 |
| REL11744 | 8,048,677 | 5,253 | 3.776 | 3.32 |
| REL11745 | 8,929,129 | 6,325 | 3.796 | 3.96 |
| REL11746 | 9,800,591 | 3,654 | 4.137 | 2.59 |
| REL11747 | 10,318,263 | 2,634 | 5.084 | 2.10 |
| REL11748 | 14,214,396 | 5,031 | 4.243 | 3.64 |
| REL11749 | 8,038,491 | 2,985 | 4.269 | 2.11 |
| REL11750 | 4,071,121 | 830 | 5.231 | 0.68 |
| REL11751 | 9,047,392 | 4,720 | 4.123 | 3.20 |
| REL11752 | 2,093,789 | 2,860 | 2.457 | 1.33 |
| REL11753 | 6,834,299 | 3,251 | 4.124 | 2.26 |
| REL11754 | 7,798,683 | 3,257 | 4.142 | 2.27 |
| REL11755 | 1,060,475 | 976 | 3.643 | 0.60 |
| REL11756 | 8,673,609 | 3,136 | 4.175 | 2.21 |
| REL11757 | 6,553,945 | 4,198 | 4.137 | 2.83 |

All twenty-four strains are clones isolated from the twelve recombination treatment populations in Souza et al. 1997, with two clones from each population.
