# Supplementary material for: Haplotype-Phased Synthetic Long Reads from Short-Read Sequencing
Source: PLoS One. 2016 Jan 20;11(1):e0147229. doi: 10.1371/journal.pone.0147229 (PMC4720449; doi:10.1371/journal.pone.0147229)
Supplement: S8 Table — (DOCX) [file pone.0147229.s025.docx]

**S8 Table.** Comparison of synthetic long read approaches for genome assembly and phasing.

|  |  | Metrics | |  |  |  |
| --- | --- | --- | --- | --- | --- | --- |
| Method | Ref. | Demonstrated N50 (kb) | Short-read bases per synthetic read base | Format | Single-tube  Multiplexing | Reagent cost per sample prep^b^ |
| This work | This work | 6.0 | 43-225 | 1-2 tubes | Demonstrated | $65 |
| TruSeq Synthetic Long Reads^a^ | 8, 13, 14 | 8.2 | 73-91 | 384-well plate | Incompatible | $848^c^ |

^a^Alternately called Moleculo or LRseq.

^b^Labor is not included in the calculations. The per-sample cost for the method described in this work is calculated assuming multiplexing of 4 samples. Further multiplexing will further reduce the cost per sample.

^c^Cost of Illumina TruSeq kit along with necessary reagents not supplied by the kit.
