# Supplementary material for: Haplotype-Phased Synthetic Long Reads from Short-Read Sequencing
Source: PLoS One. 2016 Jan 20;11(1):e0147229. doi: 10.1371/journal.pone.0147229 (PMC4720449; doi:10.1371/journal.pone.0147229)
Supplement: S9 Table — (DOCX) [file pone.0147229.s026.docx]

| **S9 Table.** Human mRNA splice variant analysis. | | | | |
| --- | --- | --- | --- | --- |
| Cell line | Total splicing junctions | Known splicing junctions | Partial novel splicing junctions (alternative 5’ or 3’) | Novel splicing junctions |
| HCT116 | 12,739 | 12,357 | 192 | 190 |
| HepG2 | 9,122 | 8840 | 129 | 153 |
