# Supplementary material for: Haplotype-Phased Synthetic Long Reads from Short-Read Sequencing
Source: PLoS One. 2016 Jan 20;11(1):e0147229. doi: 10.1371/journal.pone.0147229 (PMC4720449; doi:10.1371/journal.pone.0147229)
Supplement: S10 Table — (DOCX) [file pone.0147229.s027.docx]

**S10 Table.** Best-supported HCT116 mRNA synthetic reads spanning novel splice junctions.

| Chromosome | Intron start position | Intron stop position | No. of supporting synthetic long reads | Type^a^ |
| --- | --- | --- | --- | --- |
| 10 | 47377806 | 47387310 | 4 | Partial novel |
| 16 | 88425214 | 88425694 | 4 | Complete novel |
| 10 | 88902957 | 88903646 | 4 | Partial novel |
| 7 | 176762677 | 176764141 | 8 | Complete novel |
| 6 | 64445080 | 64447943 | 3 | Complete novel |
| 2 | 176761404 | 176762672 | 5 | Partial novel |
| 7 | 88424101 | 88425210 | 8 | Partial novel |
| 19 | 64440441 | 64444965 | 4 | Partial novel |

^a^Partial novel: alternative 5’ or 3’. Complete novel: alternative 5’ and 3’.
