# Supplementary material for: Haplotype-Phased Synthetic Long Reads from Short-Read Sequencing
Source: PLoS One. 2016 Jan 20;11(1):e0147229. doi: 10.1371/journal.pone.0147229 (PMC4720449; doi:10.1371/journal.pone.0147229)
Supplement: S11 Table — (DOCX) [file pone.0147229.s028.docx]

**S11 Table.** Best-supported HepG2 mRNA synthetic reads spanning novel splice junctions.

| Chromosome | Intron start position | Intron stop position | No. of supporting synthetic long reads | Type^a^ |
| --- | --- | --- | --- | --- |
| 2 | 73583653 | 73585593 | 4 | Complete novel |
| 2 | 85839465 | 85840338 | 4 | Partial novel |
| 4 | 73581764 | 73583644 | 6 | Complete novel |
| 5 | 99015215 | 99015503 | 4 | Complete novel |
| 3 | 145244890 | 145249544 | 4 | Complete novel |
| 5 | 223489494 | 223493388 | 4 | Partial novel |
| 2 | 99015506 | 99017013 | 4 | Complete novel |
| 3 | 1105509 | 1105663 | 4 | Complete novel |

^a^Partial novel: alternative 5’ or 3’. Complete novel: alternative 5’ and 3’.
