# Supplementary material for: Haplotype-Phased Synthetic Long Reads from Short-Read Sequencing
Source: PLoS One. 2016 Jan 20;11(1):e0147229. doi: 10.1371/journal.pone.0147229 (PMC4720449; doi:10.1371/journal.pone.0147229)
Supplement: S12 Table — (DOCX) [file pone.0147229.s029.docx]

**S12 Table.** Comparison of application categories enabled by different synthetic long read methods.

|  |  | Application category | | |
| --- | --- | --- | --- | --- |
| Method | Ref. | Genome assembly and phasing | Full-length mRNA splice variants | Phasing similar individuals (e.g. viruses) |
| This work | This work | Shown | Shown | Shown |
| BAsE-Seq | Hong et al. 2014 | Incompatible | Incompatible | Shown |
| TruSeq Synthetic Long Reads | Voskoboynik et al. 2013 | Shown | Possible | Incompatible |
