# Supplementary material for: Haplotype-Phased Synthetic Long Reads from Short-Read Sequencing
Source: PLoS One. 2016 Jan 20;11(1):e0147229. doi: 10.1371/journal.pone.0147229 (PMC4720449; doi:10.1371/journal.pone.0147229)
Supplement: S13 Table — (DOCX) [file pone.0147229.s030.docx]

| **S13 Table.** Oligonucleotides used in library preparation. | | |
| --- | --- | --- |
| Oligo # | Function | Sequence |
| Oligo 1 | Barcode adapter | 5'-/5Phos/NNN GTTCAGAGTTCTACAGTCCGACGATC NNNNNNNNNNNNNNNN CC AGGAATAGTTATGTGCATTAATGAATGG CCGC-3'  or  5'-/5Phos/NNN CCTACACGACGCTCTTCCGATCT NNNNNNNNNNNNNNNN AC AGGAATAGTTATGTGCATTAATGAATGG CCGC-3'  (a mixture of this and the above were used in the *E. coli* MG1655 experiment)  or  5'-/5Phos/NNN CCTACACGACGCTCTTCCGATCT NNNNNNNNNNNNNNNN AC AATTCCTATCGTTCACGTCGTGT CGCCATTTAGTGTCCAGTCTGA-3  (used in the *env* experiment)  or  5'-/5Phos/NNN CCTACACGACGCTCTTCCGATCT NNNNNNNNNNNNNNNN CC AGGAATAGTTATGTGCATTAATGAATGG CGCC-3’ |
| Oligo 2 | Barcode adapter, PCR primer (Rungpragayphan et al. 2002) | 5'-CCATTCAT/ideoxyU/AATGCACA/ideoxyU/ AACTATTCC/3deoxyU/G*G-3'  or  5'-CCATTCAT/ideoxyU/AATGCACA/ideoxyU/ AACTATTCC/ideoxyU/G-3'  or  5’-ACACGACG/ideoxyU/GAACGA /ideoxyU/AGGAAT/ideoxyU/G*T-3’  (used in *env* experiment) |
| Oligo 3 | lcPCR adapter | 5'-CCGAGAATTCCA*T-3' |
| Oligo 4 | lcPCR adapter | 5'-/5Phos/TGGAATTCTCGG GTGCCAAGG-3' |
| Oligo 5 | lcPCR primer | 5’-CAAGCAGAAGACGGCATACGAGAT (Index) GTGACTGGAGTT CCTTGGCACCCGAGAATTCCA-3' |
| Oligo 6 | lcPCR primer | 5'-AATGATACGGCGACCACCGAGATCTACACTCTTTCCCTACACGACGCTCTTCCGATC*T-3' |
| Oligo 7 | Barcode pairing lcPCR adapter | 5’-ACACTCTTTCCCTACACGAC GCTCTTCC-3’ |
| Oligo 8 | Barcode pairing lcPCR adapter | 5’-/5Phos/A*TC GGAAGAGC ACACGTCT |
| Oligo 9 | Barcode pairing lcPCR primer | 5’-CAAGCAGAAGACGGCATACGAGAT (Index) GTGACTGGAGTTC AGACGTGTGCTCTTCCGATC*T-3’ |
| Oligo 10 | Single-tube barcode pairing lcPCR primer | 5’-AATGATACGGCGACCACCGAGATCTACACGTTCAGAGTTCTACAGTCCGA-3’ |
| Oligo 11 | Complexity quantification | 5’-CAAGCAGAAGACGGCATACGAGAT (Index) GTGACTGGAGTTC AGACGTGTGCTCTTCCGATC CCATTCATTAATGCACATAACTATTCC-3’ |
| Oligo 12 | mRNA RT barcoding oligo-dT primer | 5′-CCATTCATTAATGCACATAACTATTCCT GGNNNNNNNNNNNNNNNN GATCGTCGGACTGTAGAACTCTGAAC T_30_VN-3′ |
| Oligo 13 | mRNA RT barcoding TSO primer | 5′- GCGGCCATTCATTAATGCACATAACTATTCCT GTNNNNNNNNNNNNNNNN AGATCGGAAGAGCGTCGTGTAGG TrGrG+G-3′ |
| Probe 1 | Quenched fluorescent qPCR probe (IDT) | 5’-/56-FAM/CCT ACA CGA /ZEN/CGC TCT TCC GAT CT/3IABkFQ/-3’ |

**Key:**

/5Phos/ = 5’ phosphate group

/ideoxyU/ = internal deoxyuracil base

/3deoxyU/ = 3’ deoxyuracil base

* = phosphorothioate linkage

rG = riboG

+G = locked nucleic acid G

N = mixture of A, T, G, and C

V = mixture of A, G, and C

T_30_ = 30 consecutive Ts

lcPCR = limited-cycle PCR

Index = 6-base Illumina TruSeq Small RNA multiplexing index sequence

/56-FAM/ = probe fluorophore

/ZEN/ = probe quencher

/3IABkFQ/ = probe quencher
