# Supplementary material for: Haplotype-Phased Synthetic Long Reads from Short-Read Sequencing
Source: PLoS One. 2016 Jan 20;11(1):e0147229. doi: 10.1371/journal.pone.0147229 (PMC4720449; doi:10.1371/journal.pone.0147229)
Supplement: S14 Table — (DOCX) [file pone.0147229.s031.docx]

| **S14 Table.** Barcode adapter oligonucleotides (Oligo 1 in S13 Table) for multiplexed library preparation. |
| --- |

| Name | Sequence |
| --- | --- |
| MULTIPLEX_RPI01 | 5’-NNN CCTACACGACGCTCTTCCGATCT NNNNNNNNNNNNNNNN ATCACG C AGGAATAGTTATGTGCATTAATGAATGG CGCC-3’ |
| MULTIPLEX_RPI02 | 5’-NNN CCTACACGACGCTCTTCCGATCT NNNNNNNNNNNNNNNN CGATGT C AGGAATAGTTATGTGCATTAATGAATGG CGCC-3’ |
| MULTIPLEX_RPI03 | 5’-NNN CCTACACGACGCTCTTCCGATCT NNNNNNNNNNNNNNNN TTAGGC C AGGAATAGTTATGTGCATTAATGAATGG CGCC-3’ |
| MULTIPLEX_RPI04 | 5’-NNN CCTACACGACGCTCTTCCGATCT NNNNNNNNNNNNNNNN TGACCA C AGGAATAGTTATGTGCATTAATGAATGG CGCC-3’ |
| MULTIPLEX_RPI05 | 5’-NNN CCTACACGACGCTCTTCCGATCT NNNNNNNNNNNNNNNN ACAGTG C AGGAATAGTTATGTGCATTAATGAATGG CGCC-3’ |
| MULTIPLEX_RPI06 | 5’-NNN CCTACACGACGCTCTTCCGATCT NNNNNNNNNNNNNNNN GCCAAT C AGGAATAGTTATGTGCATTAATGAATGG CGCC-3’ |
| MULTIPLEX_RPI07 | 5’-NNN CCTACACGACGCTCTTCCGATCT NNNNNNNNNNNNNNNN CAGATC C AGGAATAGTTATGTGCATTAATGAATGG CGCC-3’ |
| MULTIPLEX_RPI08 | 5’-NNN CCTACACGACGCTCTTCCGATCT NNNNNNNNNNNNNNNN ACTTGA C AGGAATAGTTATGTGCATTAATGAATGG CGCC-3’ |
| MULTIPLEX_RPI09 | 5’-NNN CCTACACGACGCTCTTCCGATCT NNNNNNNNNNNNNNNN GATCAG C AGGAATAGTTATGTGCATTAATGAATGG CGCC-3’ |
| MULTIPLEX_RPI10 | 5’-NNN CCTACACGACGCTCTTCCGATCT NNNNNNNNNNNNNNNN TAGCTT C AGGAATAGTTATGTGCATTAATGAATGG CGCC-3’ |
| MULTIPLEX_RPI11 | 5’-NNN CCTACACGACGCTCTTCCGATCT NNNNNNNNNNNNNNNN GGCTAC C AGGAATAGTTATGTGCATTAATGAATGG CGCC-3’ |
| MULTIPLEX_RPI12 | 5’-NNN CCTACACGACGCTCTTCCGATCT NNNNNNNNNNNNNNNN CTTGTA C AGGAATAGTTATGTGCATTAATGAATGG CGCC-3’ |
| MULTIPLEX_RPI13 | 5’-NNN CCTACACGACGCTCTTCCGATCT NNNNNNNNNNNNNNNN AGTCAA C AGGAATAGTTATGTGCATTAATGAATGG CGCC-3’ |
| MULTIPLEX_RPI14 | 5’-NNN CCTACACGACGCTCTTCCGATCT NNNNNNNNNNNNNNNN AGTTCC C AGGAATAGTTATGTGCATTAATGAATGG CGCC-3’ |
| MULTIPLEX_RPI15 | 5’-NNN CCTACACGACGCTCTTCCGATCT NNNNNNNNNNNNNNNN ATGTCA C AGGAATAGTTATGTGCATTAATGAATGG CGCC-3’ |
| MULTIPLEX_RPI16 | 5’-NNN CCTACACGACGCTCTTCCGATCT NNNNNNNNNNNNNNNN CCGTCC C AGGAATAGTTATGTGCATTAATGAATGG CGCC-3’ |
| MULTIPLEX_RPI17 | 5’-NNN CCTACACGACGCTCTTCCGATCT NNNNNNNNNNNNNNNN GTAGAG C AGGAATAGTTATGTGCATTAATGAATGG CGCC-3’ |
| MULTIPLEX_RPI18 | 5’-NNN CCTACACGACGCTCTTCCGATCT NNNNNNNNNNNNNNNN GTCCGC C AGGAATAGTTATGTGCATTAATGAATGG CGCC-3’ |
| MULTIPLEX_RPI19 | 5’-NNN CCTACACGACGCTCTTCCGATCT NNNNNNNNNNNNNNNN GTGAAA C AGGAATAGTTATGTGCATTAATGAATGG CGCC-3’ |
| MULTIPLEX_RPI20 | 5’-NNN CCTACACGACGCTCTTCCGATCT NNNNNNNNNNNNNNNN GTGGCC C AGGAATAGTTATGTGCATTAATGAATGG CGCC-3’ |
| MULTIPLEX_RPI21 | 5’-NNN CCTACACGACGCTCTTCCGATCT NNNNNNNNNNNNNNNN GTTTCG C AGGAATAGTTATGTGCATTAATGAATGG CGCC-3’ |
| MULTIPLEX_RPI22 | 5’-NNN CCTACACGACGCTCTTCCGATCT NNNNNNNNNNNNNNNN CGTACG C AGGAATAGTTATGTGCATTAATGAATGG CGCC-3’ |
| MULTIPLEX_RPI23 | 5’-NNN CCTACACGACGCTCTTCCGATCT NNNNNNNNNNNNNNNN GAGTGG C AGGAATAGTTATGTGCATTAATGAATGG CGCC-3’ |
| MULTIPLEX_RPI24 | 5’-NNN CCTACACGACGCTCTTCCGATCT NNNNNNNNNNNNNNNN GGTAGC C AGGAATAGTTATGTGCATTAATGAATGG CGCC-3’ |

Multiplexing index regions are underlined.
